# A comparative meta-proteomic pipeline for the identification of plasmodesmata proteins and regulatory conditions in diverse plant species

**DOI:** 10.1186/s12915-022-01331-1

**Published:** 2022-06-02

**Authors:** Philip Kirk, Sam Amsbury, Liam German, Rocio Gaudioso-Pedraza, Yoselin Benitez-Alfonso

**Affiliations:** 1 Centre for Plant Science, School of Biology, University of Leeds, Leeds, LS2 9JT UK; 2 Plants, Photosynthesis and Soil, School of Biosciences, University of Sheffield, Sheffield, S10 2TN UK

**Keywords:** Cell-to-cell communication, Plasmodesmata located proteins, Symplasmic transport, Transcriptomic analysis, *Arabidopsis thaliana*, *Medicago truncatula*, Callose, Osmotic root responses, Nitrogen-fixing symbiosis

## Abstract

**Background:**

A major route for cell-to-cell signalling in plants is mediated by cell wall-embedded pores termed plasmodesmata forming the symplasm. Plasmodesmata regulate the plant development and responses to the environment; however, our understanding of what factors or regulatory cues affect their structure and permeability is still limited. In this paper, a meta-analysis was carried out for the identification of conditions affecting plasmodesmata transport and for the in silico prediction of plasmodesmata proteins in species for which the plasmodesmata proteome has not been experimentally determined.

**Results:**

Using the information obtained from experimental proteomes, an analysis pipeline (named plasmodesmata in silico proteome 1 or PIP1) was developed to rapidly generate candidate plasmodesmata proteomes for 22 plant species. Using the in silico proteomes to interrogate published transcriptomes, gene interaction networks were identified pointing to conditions likely affecting plasmodesmata transport capacity. High salinity, drought and osmotic stress regulate the expression of clusters enriched in genes encoding plasmodesmata proteins, including those involved in the metabolism of the cell wall polysaccharide callose. Experimental determinations showed restriction in the intercellular transport of the symplasmic reporter GFP and enhanced callose deposition in Arabidopsis roots exposed to 75-mM NaCl and 3% PEG (polyethylene glycol). Using PIP1 and transcriptome meta-analyses, candidate plasmodesmata proteins for the legume *Medicago truncatula* were generated, leading to the identification of Medtr1g073320, a novel receptor-like protein that localises at plasmodesmata. Expression of Medtr1g073320 affects callose deposition and the root response to infection with the soil-borne bacteria rhizobia in the presence of nitrate.

**Conclusions:**

Our study shows that combining proteomic meta-analysis and transcriptomic data can be a valuable tool for the identification of new proteins and regulatory mechanisms affecting plasmodesmata function. We have created the freely accessible pipeline PIP1 as a resource for the screening of experimental proteomes and for the in silico prediction of PD proteins in diverse plant species.

**Supplementary Information:**

The online version contains supplementary material available at 10.1186/s12915-022-01331-1.

## Background

Plants have evolved a myriad of long- and short-distance signalling pathways underpinning their ability to adapt and thrive in diverse conditions on Earth. A major route for signalling is the symplasm: the continuous cytoplasmic connections established by cell wall-embedded pores termed plasmodesmata (PD). PD are dynamic structures tightly controlled to regulate intercellular signalling during development and in response to the environment [1]. Despite their importance, outstanding questions remain regarding the molecular composition and the conditions that affect PD function.

Over the last decade, proteomic analysis of PD-enriched membrane fractions has greatly improved the identification of proteins that localise to or associate with PD [2–5]. Localisation of labelled protein fusions using confocal, FRET-FLIM (Förster resonance energy transfer by fluorescence lifetime imaging), or transmission electron microscopy has experimentally confirmed PD association for around 60 proteins in the model plant *Arabidopsis thaliana* (Additional file 1: Table S1) [2, 5–33]. These include CALLOSE SYNTHASE 3 (CALS3) [13], the PD-located β-(1,3)–GLUCANASES (PdBGs and AtBG_PPAP) [26, 34] and the PD-CALLOSE BINDING proteins (PDCBs) [18]. It also comprises signalling proteins and kinases such as the PD-LOCATED PROTEINs (PDLPs) and the LYSIN MOTIF DOMAIN-CONTAINING GLYCOSYLPHOSPHATIDYLINOSITOL-ANCHORED PROTEIN 2 (LYM2) [25, 28]. Proposed functions for many of these factors was recently reviewed in the context of cell-cell connectivity [35, 36]. Many of these activities participates in a mechanism that regulates symplasmic transport by controlling the synthesis/degradation of the β-(1,3)–glucan callose at PD surrounding cell walls [36]. Specifically, members of the PDLP family modify callose metabolism in response to elicitors, viruses, bacterial and fungal infections [37–40].

Identification in the PD proteomes is not a proof of PD localisation as preparations are often contaminated with non-PD structures such as the plasma membrane (PM), cell walls and the endoplasmic reticulum (ER) [5]. The presence of ER proteins was attributed to the desmotubule (DT): a tubular structure that runs through PD connecting the ER of neighbouring cells. To eliminate contaminants, PD-enriched fractions (i.e. detergent-resistant microsomal fractions) were generated and their proteomes were compared to other membrane proteomes [4, 5]. This is far from an ideal solution, especially considering the discovery of PM proteins that transiently localise at PD regions in response to stress conditions [22, 27]. Another limitation is that, so far, all PD proteomes are isolated from cell cultures; thus, identification of proteins involved in dynamically regulating complex or secondary PD (formed post-cytokinesis) is missed. The majority of the verified PD proteins belong to large multigene families; thus, phylogenetic analysis is often used to identify family members in a differentiated tissue or orthologues in different plants but applying this approach on a gene-by-gene basis is time-consuming and not always successful [41, 42]. A recent success using this approach led to the identification of MtBG2, a β-(1,3)–glucanase in *Medicago truncatula* that participates in callose degradation and regulates symplasmic transport and the initiation of nodules harbouring the nitrogen-fixing bacteria rhizobia [42]. This and other studies support the existence of conserved domains or signatures associated to PD localisation.

In this paper, we present a meta-analysis that exploits the conserved structural features of verified PD proteins in the generation of in silico proteomes for species in which experimental data is not available. Comparing four published proteomes, we observed considerable overlap in protein family and subfamily composition. Based on this, we developed an analysis pipeline (implemented in R and named Plasmodesmata In silico Proteome 1 or PIP1 [43]) to rapidly generate candidate PD proteome lists for species annotated in both Ensembl Plant [44] and PANTHER16 [45] databases (currently 22 species, Additional file 1: Table S2). Transcriptomic data allowed us to generate co-expression tables (interactomes) reflecting interactions, conditions and genes involved in the molecular mechanisms affecting PD function. Using this approach, we identified salinity and osmotic stress as conditions regulating callose deposition and symplasmic transport. We also identify a cluster of genes co-expressed with *PDLPs* and *AtBG_PPAP* likely involved in PD and cell wall regulation in response to these stresses. Furthermore, an in silico PD proteome was obtained for *M. truncatula* which identified a gene (Medtr1g073320) co-expressed with MtBG2 in response to rhizobia infection. Transgenic expression of Medtr1g073320 (fused to YFP) confirmed its PD localization and reveal its role in regulating callose and the root response to rhizobia infection in nitrate replete media. We discuss the advantages of using PIP1 in the screening of experimentally obtained proteomes, in the identification of novel PD proteins in species in which proteomes are not available and in identifying conditions affecting PD function, thus intercellular signalling and plant development.

## Results

### Workflow for the prediction of PD proteomes in silico and for the screening of contaminants in experimental proteomes

**Fig. 1:**
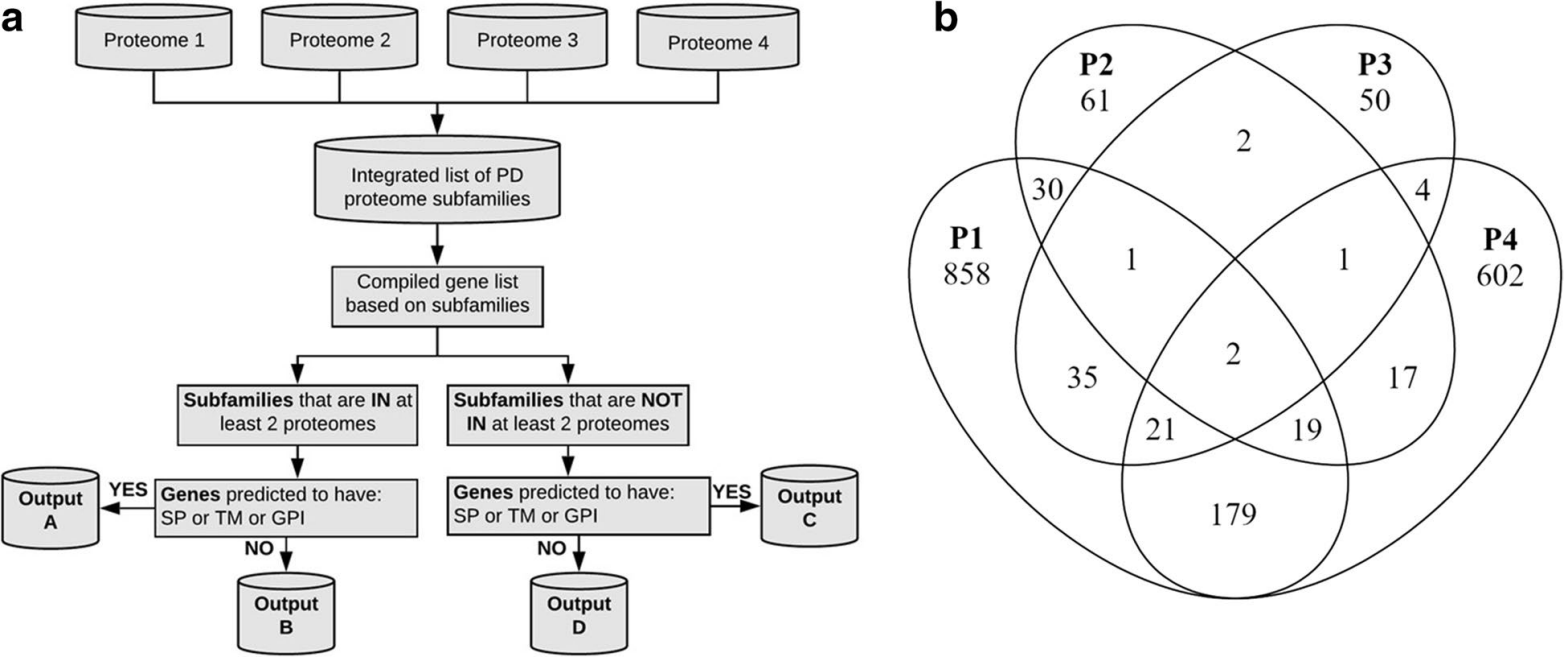
Pipeline to determine candidate plasmodesmata (PD) proteome. **a** A flowchart explaining PIP1 approach is presented. Proteome data, determined experimentally by [2] (Proteome 1, P1), [3] (Proteome 2, P2), [5] (Proteome 3, P3) and [4] (Proteome 4, P4), were used. A list was compiled of subfamilies of proteins identified in P1-P4 using PANTHER16. Genes belonging to these subfamilies were extracted for the target plant species. These were classified by whether its subfamily was present in one or in two or more experimental proteomes. Predictions of encoding a signal peptide (SP), transmembrane domain (TM) or glycophosphatidylinositol anchor (GPI) were used to classify the genes as these features are enriched in verified PD proteins (see Additional file 1: Table S1). PIP1 outputs 4 list of genes (A-D) according to these classifications representing the in silico PD proteome for the target plant. **b** A Venn diagram showing the overlap between subfamilies identified in P1-P4 experimental proteomes (311 subfamilies appear in more than one proteome). See Additional file 1: Table S3

Proteomic data obtained from PD-enriched fractions extracted from *A. thaliana* [2, 5], *Nicotiana benthamiana* [3] and *Populus trichocarpa* [4] cell cultures were introduced into a custom-built R-based pipeline released as a resource with this article [43]. The workflow is described in Fig. 1a and in the “Methods” section. The main idea emerges from the substantial overlap observed in gene subfamilies identified in independent PD proteomes, with 311 subfamilies identified in more than one proteome (Fig. 1b, Additional file 1: Table S3). We used subfamily classifications (annotated in PANTHER16 [45]) to predict the genes/proteins forming the in silico proteome for a particular plant of interest.

The pipeline categorises the output by whether the subfamily is present in one or multiple PD proteomes and based on predictions of distinctive features identified in PD-verified genes (Fig. 1). These features were predicted for a list of PD genes with previously verified localization in *A. thaliana* (Additional file 1: Table S1) using multiple online platforms as described in Methods [46–50]. When compared to the whole Arabidopsis proteome, PD-localised proteins are overrepresented in predicted signal peptide (SP), transmembrane domain (TM), glycophosphatidylinositol anchor (GPI), s-geranylgeranylation and s-palmitoylation (Additional file 2: Figure S1). The presence of a SP in combination with either a GPI and/or TM domain returned the highest proportion of verified genes; thus, these features were chosen for gene categorisation.

The pipeline outputs four lists of genes/proteins. PIP1-A are genes encoding proteins from subfamilies present in more than one PD proteome with a predicted SP, GPI or TM domains. PIP1-B are also present in multiple proteomes but lacking the membrane localising features. PIP1-C and PIP1-D are proteins in subfamilies found in a single proteome either with predicted targeting features (PIP1-C) or without them (PIP1-D) (Fig. 1). As PD verified proteins usually display membrane-targeting features, PIP1-A and PIP1-C are the most likely candidates. In support, these lists contain subfamilies of genes encoding known PD activities such as enzymes involved in callose metabolism (e.g. BG and CALS) and signalling (e.g. PDLPs) (Additional file 1: Table S1). The main strength of the pipeline is that it also identifies subfamilies not previously characterised as having PD localization despite some members being isolated in multiple experimental proteomes. Additional file 1: Table S3 summarises these results and should be used to prioritise the characterisation of new candidates based on subfamily counts in experimental proteomes.

**Fig. 2:**
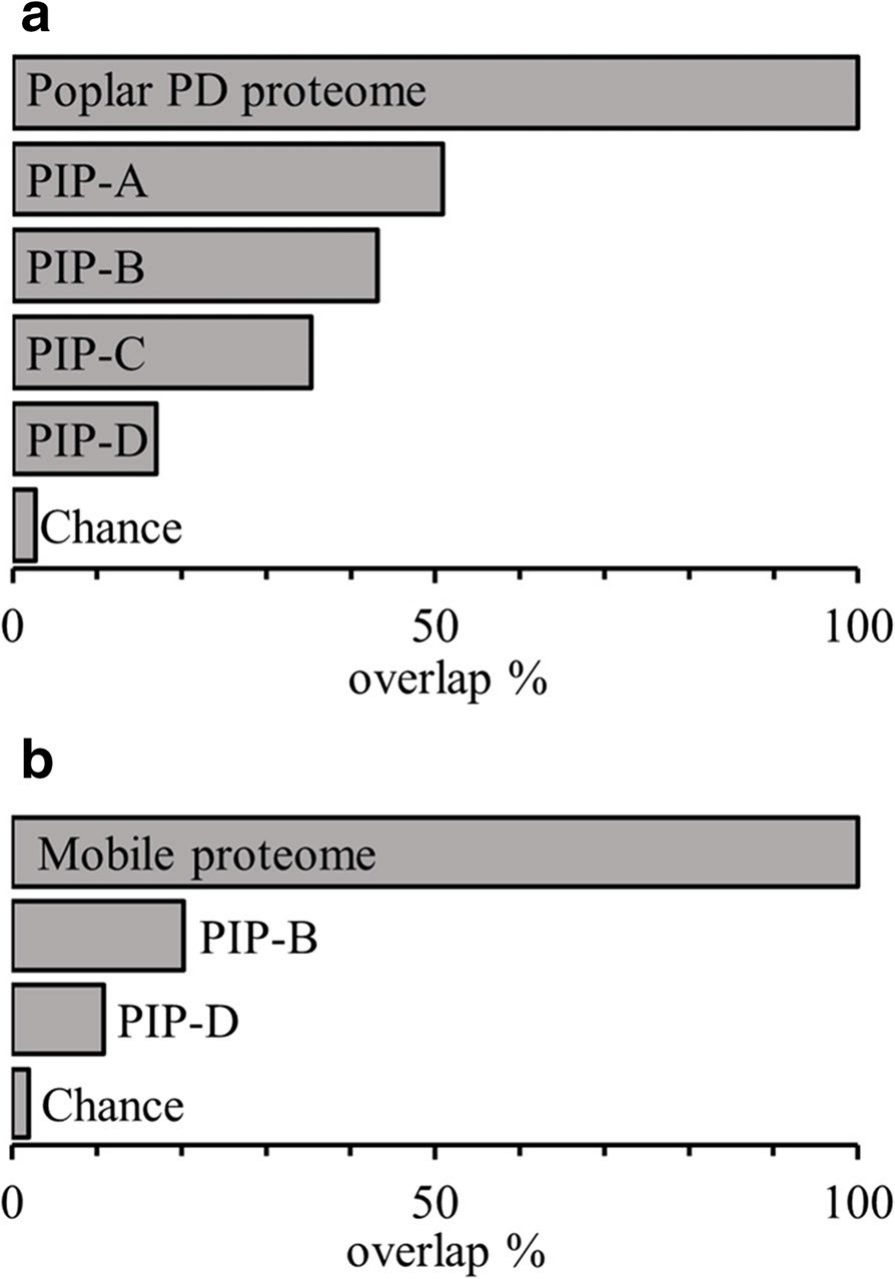
Overlaps between PIP1 outputs and experimental proteomes. In silico proteome PIP1-A-D for poplar and *Arabidopsis thaliana* were generated using the pipeline described in Fig. 1 (see Additional file 1: Table S4 and Table S5). **a** Overlap (in %) between the in silico and the experimental poplar proteome determined in [4]. See also Additional file 2: Fig. S2. **b** Overlap (in %) between PIP1-B and PIP1-D lists and the mobile proteome reported for *A. thaliana* in [51]. In both cases, the size of the overlap by chance was determined by bootstrap sampling of the whole genome (10,000 cycles, median % overlap given)

To evaluate PIP1 predictive power, we generated the in silico proteome for *P. trichocarpa* (Additional file 1: Table S4) and compared the results with the published raw experimental proteome [4]. 1032 out of 1148 experimentally determined proteins were predicted by PIP1, and 50% identified in PIP1-A (Fig. 2a). One hundred sixteen proteins identified in the experimental proteome are not annotated within PANTHER16 subfamilies, thus are not predicted by PIP1. When the poplar proteome is excluded as input, the overlap remains 20 times larger than would be expected by chance (Additional file 2: Figure S2).

To further evaluate the pipeline, the Arabidopsis in silico proteome was investigated. PIP1 outputs 206 Arabidopsis genes (158 subfamilies) in PIP1-A, 208 genes (152 subfamilies) in PIP1-B, 751 genes (597 subfamilies) in PIP1-C and 1117 genes (802 subfamilies) in PIP1-D (Additional file 1: Table S5). Candidate lists A and C contain mostly predicted secreted proteins including 36 proteins verified to localise at PD based on multiple publications (Additional file 1: Table S1). These include the AtBG_PPAP [34], the MULTIPLE C2 DOMAIN AND TRANSMEMBRANE REGION PROTEINs, MCTP6 and MCTP9 [5] and the leucine-rich repeat receptor-like serine/threonine-protein kinase BARELY ANY MERISTEM 2, BAM2 [24].

Despite lacking predicted membrane-targeting features, list B was identified in multiple proteomes and contains REMORIN 1.2 (REM1.2) [29], which is a peripheral membrane PD protein. PIP1-D were identified in a single proteome, thus are likely contaminants but list B may also contain mobile proteins unintentionally captured within PD fractions while being transported. To test this hypothesis, the candidate PD gene lists were compared to the mobile proteome identified in *Cuscuta australis* (dodder) parasitising *A. thaliana* [51]. There was significant overlap between the mobile proteome and both lists B and D (Fig. 2b, Additional file 1: Table S5). PIP1-B had the largest over-representation (20.2%) with an overlap over 10x greater than would be expected by chance.

In summary, we developed PIP1 as a new resource to generate candidate PD proteomes based on subfamily annotation and membrane-targeting structural features. The pipeline is compatible with 22 plant species annotated in PANTHER16 (Additional file 1: Table S2), and as demonstrated for poplar, it can effectively predict raw experimental proteomes. PIP1-A and PIP1-C lists contain secreted proteins and are the most likely PD components. As shown for Arabidopsis, the PIP1-B list contains mobile and peripheral membrane proteins whereas PIP1-D are likely contaminants isolated in experimental proteomes. The pipeline serves as a resource to prioritise candidates for experimental verification based on the number of proteomes where the subfamily is identified and predictions on subcellular localization and membrane targeting features.

### Interactome networks identify salinity and drought as PD regulatory conditions

**Fig. 3:**
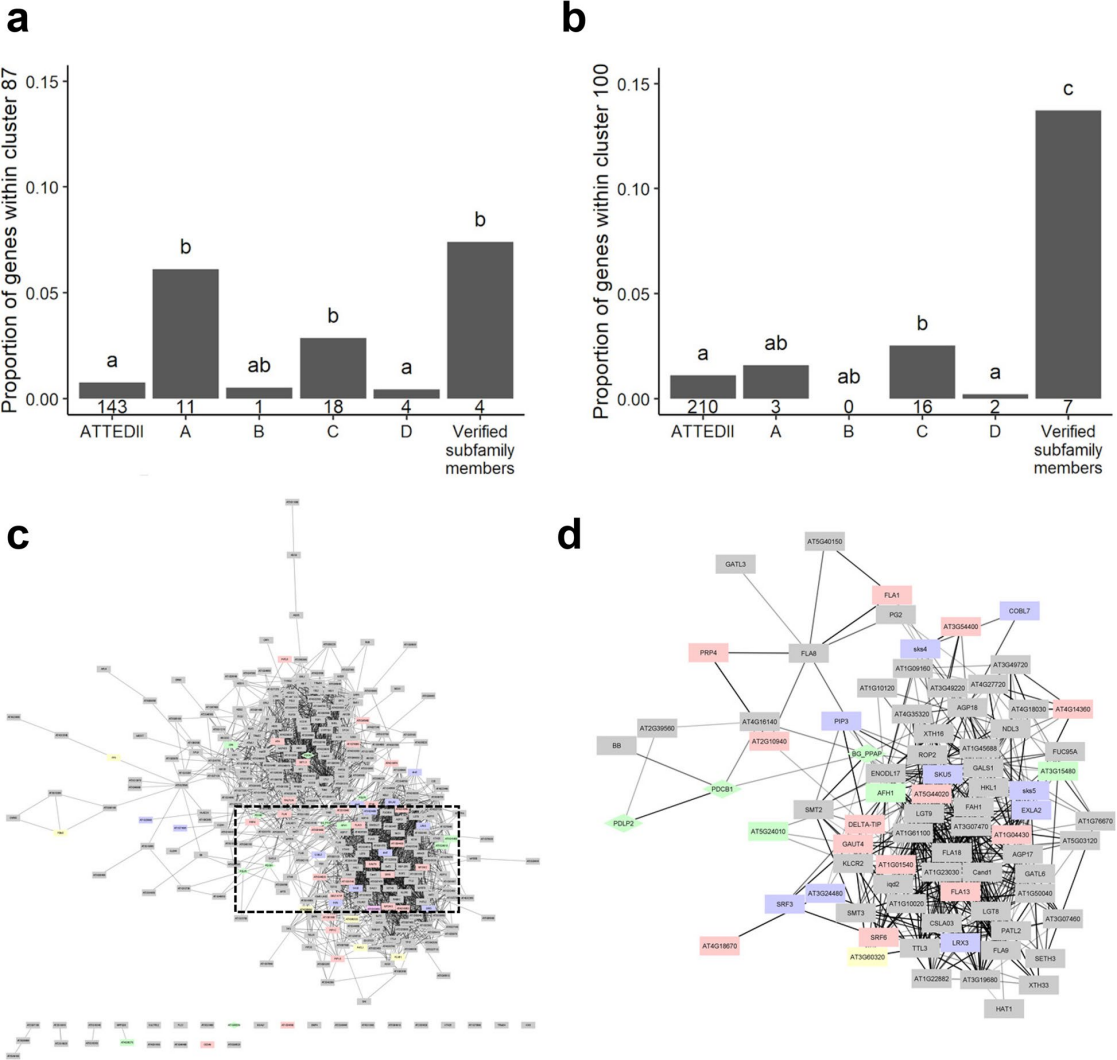
Co-expression analysis between PIP1 candidates and PD verified genes. *A. thaliana* co-expression data was extracted from ATTED-II database [52] and clustered based on hierarchical clustering (k = 151). **a** Cluster 87 and **b** cluster 100 show significant over-representation in verified subfamily members, PIP1-A and PIP1-C in relation to the whole ATTED-II database. Number underneath each bar = number of genes included within that cluster. Bars with differing letters above are significantly different (Fisher’s exact test, Holm corrected, *p* ≤ 0.05). **c** Clusters 87 and 100 represented using Cytoscape [53] where edge opacity was set to represent the degree of correlation between genes. Correlations <5.0 were filtered out. Node colour was set to represent candidates in PIP1-A (purple), PIP1-C (pink) and verified PD genes (green). Rhomboid shape are genes encoding proteins related with callose metabolism (manually curated). **d** Sub-network generated by selecting ‘first and second neighbours’ of callose- regulators BG_PPAP, PDLP2 and PDCB1 which map within the interactome region marked with discontinuous lines in **c**. Genes are listed in Additional file 1: Table S6

We explore the potential use of PIP1 in identifying conditions that regulate PD function. We used hierarchical clustering to organise PIP1 outputs based on their expression profiles in the ATTED-II database [52] as described in the ‘Methods’ section. For *A. thaliana*, we identified two clusters significantly over-represented in PD-verified proteins relative to the wider database: cluster 87 (Fig. 3a) and cluster 100 (Fig. 3b). These clusters contained more than a hundred genes including 14 genes in PIP1-A and 34 from PIP1-C (Fig. 3c).

Callose is a key regulator of PD transport and proteins involved in the synthesis, and degradation of callose is represented in these co-expression clusters. Figure 3d shows the nearest 1st and 2nd order neighbours of the PD-callose regulatory proteins BG_PAPP, PDCB1 and PDLP2 identified in clusters 87 and 100 (Additional file 1: Table S6). The network also displays genes indirectly associated with callose regulation such as an the *SKU5-SIMILAR* family (*SKS*) and of the *STRUBBELIG-Receptor Family* (*SRF*s). This interactome also includes genes encoding cell wall activities that might co-exist with callose with the potential to regulate PD such as *XYLOGLUCAN ENDOTRANSGLUCOSYLASE/HYDROLASEs (XTH)*, *POLYGALACTURONASE 2 (PG2)*, *GALACTURONOSYLTRANSFERASEs (GAUTs)* and *ARABINOGALACTAN PROTEINs* (*AGPs* and fasciclin-like-*AGP*s). The network also displays proteins with a role in PD callose regulation such as BG_PAPP, PDCB1 and PDLP2 and several members of the SKU5-like family (SKS) and of the STRUBBELIG-receptor family (SRFs) which are indirectly associated with this mechanism. Genes identified in these interactomes, especially those belonging to PIP1-A, are good candidates for the discovery of novel PD proteins.

**Fig. 4:**
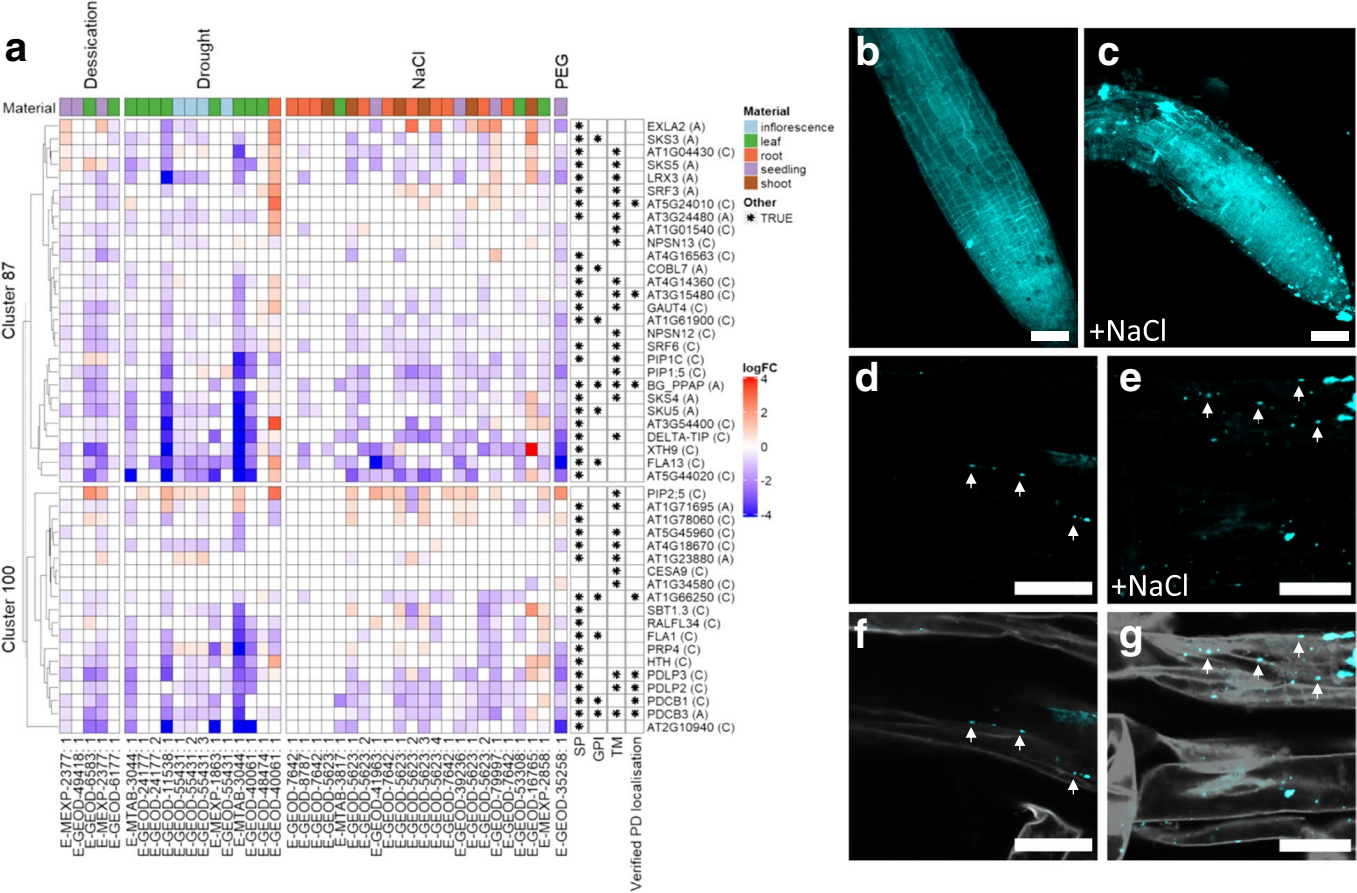
The expression of PD candidates and callose deposition is regulated in response to osmotic and salinity stress. **a** Differential expression (log_2_FC) of genes in PIP1-A, PIP1-C and verified PD tables grouped in clusters 87 and 100 (see Additional file 1: Table S6) was determined using public microarrays (see Additional file 1: Table S7 and Additional file 2: Figure S3). Profiles range from blue to red (downregulated to upregulated in relation to control). Column labels at the bottom show ArrayExpress accession codes followed by a reference number as listed in Additional file 1: Table S7 [54]. Rows are ordered by hierarchical clustering (dendrogram). Asterisks in cells on the right denote predictions on membrane targeting features or verified PD localization. SP = Signal peptide, GPI = glycophosphatidylinositol anchor, TM = transmembrane domain. Cell colour above each column represents the material sampled as indicated in the legend. **b-g** Aniline blue staining of 7 days old Arabidopsis roots grown on ATS (**b, d, f**) or ATS with 75mM NaCl (**c, e, g**) media. The pictures are confocal images of root tips (**b-c**) and high magnification sections in the differentiation zone (**d-g**) of roots stained with aniline blue (405 nm, blue; **d-e**) and with propidium iodide (561 nm, shown in grey; **f-g**). Arrows in **d-e** show punctate pattern of callose in cell walls reminiscent of PD. The pictures are representative of at least three independent biological replicas (see Additional file 2: Figure S4). Scale bar (**b-c**) = 50 μm; (**d-g**) = 20 μm

To understand the conditions regulating clusters 87 and 100, publicly available microarrays [54] were re-analysed as described in the ‘Methods’ section. Drought, desiccation, elevated NaCl and polyethylene glycol (PEG) were found as conditions strongly regulating candidate genes expression (Fig. 4a and Additional file 2: Figure S3). To determine if changes in gene expression reflect conditions affecting PD regulation, we tested the deposition of callose and symplasmic transport, in Arabidopsis roots exposed to elevated NaCl and PEG.

Seedlings were germinated in *Arabidopsis thaliana* salts (ATS) media (control conditions), ATS supplemented with 3% PEG or with 75mM NaCl. At 6 days post germination (dpg), roots were stained with aniline blue which reveals callose as fluorescent deposits under the ultraviolet (UV) light. In control roots, callose accumulates at the cell plates in the root meristem but deposits (or aggregates) were found in roots treated with 75mM NaCl (Fig. 4b–g). Closer visualisation of roots co-stained with aniline blue and propidium iodide (which stains cell walls) indicates that callose excessively accumulates at cell walls in roots grown in PEG and NaCl, in a punctate pattern reminiscent of PD (Fig. 4d–g, see also Additional file 2: Figure S4). To determine if callose is directly associated with the root response to salt and osmotic stress, we phenotypically characterised root growth in Arabidopsis seedlings overexpressing PDLP1 (PDLP1OE) [28], described to induce callose. PDLP family members were identified in the network interactome analysis and are regulated in response to osmotic stresses (Figs. 3 and 4). Root growth was restricted in PDLP1OE in relation to wild-type (WT) grown in control conditions (Additional file 2: Figure S5). Exposure to 75-mM NaCl (which reduces water potential from −0.66 to −1 MPa) further reduces root length in both WT and PDLP1OE. We also determined root length of plants in plates containing 3% PEG, as a milder osmotic stress (water potential ~ −0.9MPa). PEG addition reduced root growth in WT but PDLP1OE inhibits this response and roots show similar phenotype as in control conditions. This result suggests that constitutive PDLP1 expression primes root response to mild osmotic stress conditions.

**Fig. 5:**
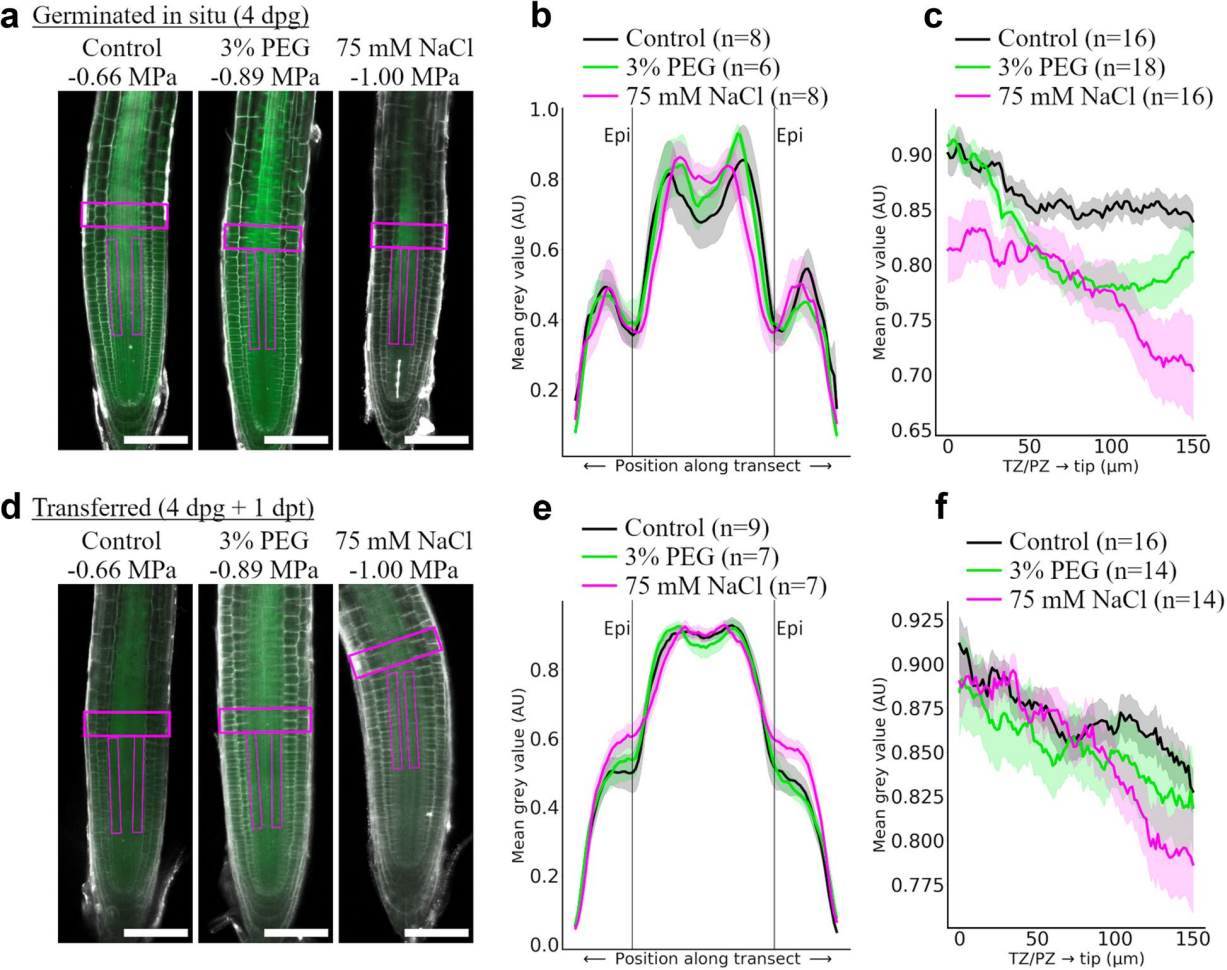
GFP diffusion in the root meristem of *Arabidopsis thaliana* is restricted in 3% PEG and 75 mM sodium chloride. **a** Seeds expressing p*SUC2*::GFP were grown on ATS control media, ATS supplemented with 3% polyethylene glycol (PEG) or with 75 mM NaCl. Roots were collected at 4 days post-germination (4 dpg) and counterstained with propidium iodide (PI). Images were sequentially collected at 561 nm (PI shown in grey) and 488 nm (green: GFP). Estimated water potential (MPa, see main text) is labelled on top of the representative pictures. **b**, **c** Transects highlighted by magenta boxes in (**a**) were used to determine GFP fluorescence profiles using mean grey values (AU: arbitrary units). **b** shows transects across the transition zone (TZ) while (**c**) shows transects rootward starting from the transition/proliferation zone (TZ/PZ) towards the root tip. **d-f** Similar experiments were carried out with seedlings germinated on ATS media and transferred for 1 day (1dpt) to 3% PEG or 75 mM NaCl. Scale bar = 100 μm. Charts show traces representing aggregate min-max normalised GFP fluorescence along the transect (mean ± SE, N indicated in the key)

To determine the effect on symplasmic communication, we used transgenic *A. thaliana* seedlings expressing GFP, driven by the phloem companion cell-specific promoter *Sucrose Symporter 2 SUC2* (p*SUC2*::GFP) [55]. p*SUC2*::GFP seedlings were grown in either ATS control, 75-mM NaCl or 3% PEG. In control media, GFP symplasmically diffuses out of the phloem spreading throughout the whole root meristem (Fig. 5). Changes in GFP distribution were measured in roots grown for 4 days directly on PEG or on NaCl media (Fig. 5a) and in roots grown in ATS and exposed to PEG or NaCl media for 24h (Fig. 5d). The mean grey values in ImageJ measured lateral and rootward diffusion of GFP from confocal images (Fig. 5). Transversal profiles, measured in the transition/elongation zone, showed no major difference in lateral distribution of GFP in both experiments (chart traces representing min-max fluorescence for *N* >6 overlaps) (Fig. 5b, e). In contrast, the rootward profiles show a decrease in fluorescence in both, 3% PEG and 75mM NaCl (Fig. 5c, f). Coincident with callose deposition and a lower water potential (Fig. 4), the effect is more apparent in NaCl, particularly between 50 and 150 μm towards the tip (rootward fluorescence profiles) where relative fluorescence drops over 13% after germination in 75-mM NaCl (Fig. 5c) and decreased 11% in plants transferred to this media for 24h (Fig. 5f).

**Fig. 6:**
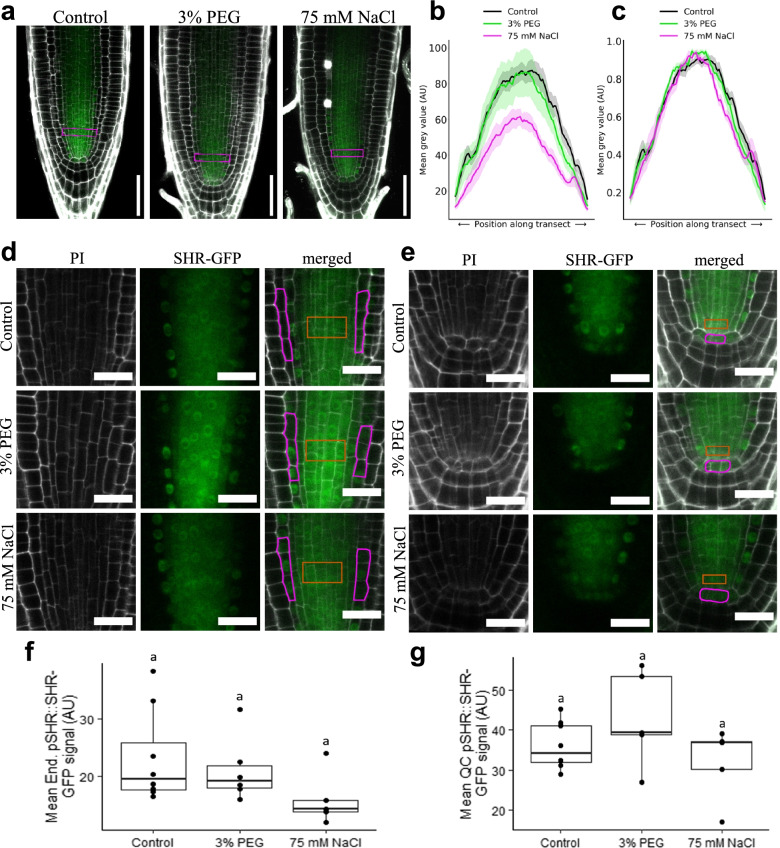
SHR-GFP expression is reduced in the root meristem of *Arabidopsis thaliana* grown in 75 mM sodium chloride. Seeds expressing p*SHR*::SHR-GFP were grown on ATS control media, ATS supplemented with 3% polyethylene glycol (PEG) or with 75 mM NaCl. Roots were collected at 6 days post-germination and counterstained with propidium iodide (PI). Images were sequentially collected at 561 nm (PI shown in grey) and 488 nm (green: GFP). **a** Representative pictures showing primary root meristems. Transects highlighted by magenta boxes were used to determine GFP fluorescence profiles using mean grey values (AU: arbitrary units). **b, c** Charts show lateral profiles 30 μm from the QC​, **b** show non-normalised green fluorescence values whereas (**c**) show traces representing min-max normalised fluorescence along the transect for N=6 plants (mean ± SE). **d-e** Representative pictures of root meristems grown in the three media (control, 3% PEG and NaCl) showing PI channel (in grey), GFP (green) and a picture of the channels superimposed. SHR is expressed in the stele (orange boxes) and move into the endodermis and QC, highlighted by magenta boxes. Pictures in **d** show movement to the endodermis whereas **e** shows movement to the QC region. Panels **f** and **g** show quantification of the GFP fluorescence in the endodermis and QC respectively in, at least, 5 independent roots (*N*=5). Box plots display range, interquartile range, median and individual data points are marked with black circles. No significant difference between treatments was observed (One-way ANOVA, Tukey post-hoc test, *p* ≤ 0.05). Scale bar **a**= 50 μm; **d-e**= 20 μm

To evaluate the effect of osmotic conditions on developmental proteins, intercellular transport of the transcription factor SHORTROOT (SHR) was measured. SHR cDNA is expressed in the stele, but the protein actively moves into the endodermis and the quiescent centre (QC) [56]. Roots expressing p*SHR*::SHR-GFP were grown on ATS, ATS + 3% PEG and ATS + 75mM NaCl. GFP fluorescence was imaged using confocal microscopy at 6 dpg, and fluorescence values were quantified in a transect across the endodermis and pro-vascular tissue (Fig. 6a–c). Exposure to NaCl significantly reduced SHR expression, but when profiles are normalised, no significant difference in protein distribution was observed. A closer look at SHR expression in the endodermis and the QC indicates that the protein accumulates in the nuclei of endodermal cells in all tested conditions, but the amount of protein is reduced in the QC of roots exposed to NaCl (Fig. 6d–g).

Taken together, the data suggest that network expression analysis of PIP1 can be used to identify conditions affecting PD regulation and reveal gene candidates potentially involved in the underlying mechanism. Salinity and osmotic stress regulate PD verified genes and PIP1 candidates, which aligns well with the changes in callose and symplasmic connectivity observed in Arabidopsis roots treated with NaCl and PEG.

### PIP1 identifies Medtr1g073320 which regulates callose and nitrate-dependent response to rhizobia in Medicago truncatula

PIP1 was designed to predict proteomes in species in which experimental data is not available. This is the case for all legumes including *Medicago truncatula*. Using the workflow described in Fig. 1, the pipeline generated the first in silico proteome for *M. truncatula* (Additional file 1: Table S8). Lists A and C comprised 1018 genes belonging to subfamilies represented in at least one experimental proteome and displaying membrane targeting features (SP, GPI or TM). These lists include orthologues for the CALS, PDCB and PDLP genes.

**Fig. 7:**
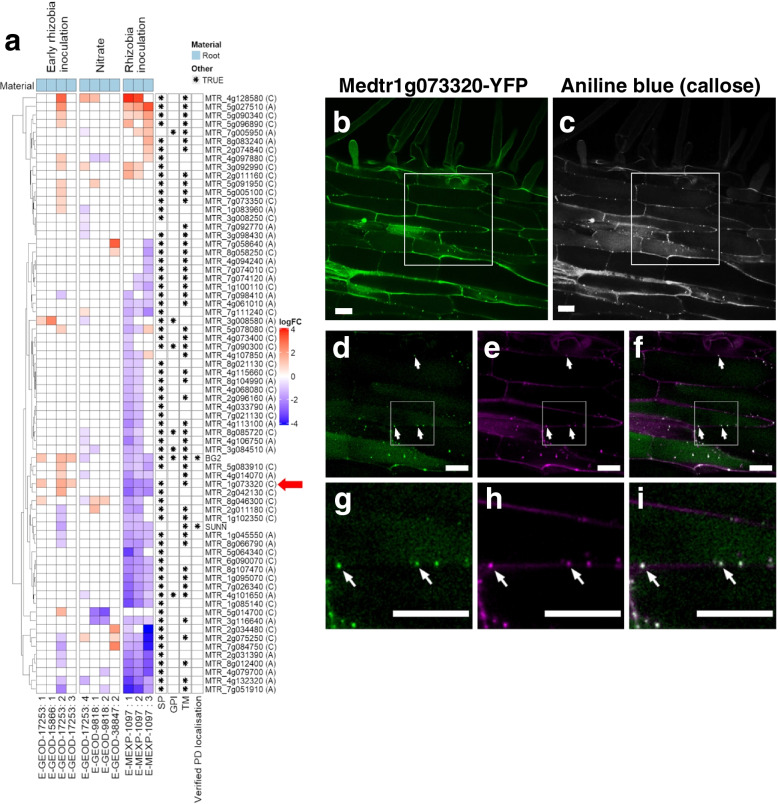
The *Medicago truncatula* protein Medtr1g073320 is regulated in response to rhizobia and co-localizes with callose at plasmodesmata. The in silico proteome PIP1 for *Medicago truncatula* was generated using the pipeline (see Additional file 1: Table S8). **a** Expression analysis of PD candidate genes isolated in PIP1-A and PIP1-C co-regulated with SUNN and MtBG2 in response to nitrate and rhizobia inoculation. Differential gene expression (log_2_FC) was determined using public microarray data of experiments relating to nitrate and rhizobia inoculation in root tissue (see Additional file 1: Table S7 for information on selected microarrays). Expression profiles range from blue to red (downregulated to upregulated in relation to control). Column labels at the bottom show ArrayExpress accession codes followed by a reference number [54]. Rows are ordered by hierarchical clustering (dendrogram on the left). Asterisks in cells on the right denote predictions on membrane targeting features or verified PD localization. SP = Signal peptide, GPI = glycophosphatidylinositol anchor, TM = transmembrane domain. Red arrow indicates the position of MTR_1g073320. **b-i** Confocal microscope images of roots expressing Medtr1g073320 fused with YFP (green) counterstained with aniline blue (grey in **c**, magenta in **e, h**) to reveal callose. Images shown in **b, d,** and **g** were obtained with excitation laser 561 nm (YFP). Images shown in **c, e** and **h** were obtained using excitation laser 405 nm (aniline blue). Merged images are shown in (**f**) and (**i**). Co-localization events are highlighted with arrows. Panels **d-f** are a magnification of the area highlighted by the white box in (**b-c**). Panels **g-i** are magnification of the area highlighted in the panels **d-f**. Scale bar = 20 μm. See also Additional file 2: Fig. S7

Previous research identified that the *M. truncatula* β-(1,3)-GLUCANASE, MtBG2 [42] and the SUPER NUMERIC NODULES (SUNN) protein [57] both localise at PD. Both proteins regulate nodulation upon infection with the symbiotic bacteria rhizobia. We tested the use of PIP1 combined with transcriptomics (as described for *A. thaliana)* for the identification of PD proteins coregulated with MtBG2 and SUNN in the root response to rhizobia. The expression of *M. truncatula* genes in PIP1-A and PIP1-C was studied in microarrays corresponding to early rhizobia inoculation, nitrate treatments and during nodulation (Fig. 7). A cluster containing MtBG2 and SUNN was identified comprising 70 genes from list A and C (Fig. 7a). Genes in this cluster share a similar expression pattern, particularly in roots inoculated with rhizobia (E-MEXP-1097, [54]). Four genes show a strikingly similar expression profile to MtBG2. These are MTR_5g083910, MTR_4g014070, MTR_1g073320 and MTR_2g011180.

Phylogenetic and sequence analysis identified MTR_1g073320 (gene ID Medtr1g073320) as a receptor-like kinase closely related to PDLP2 and PDLP3 (Additional file 2: Figure S6). PDLPs regulate callose and microbial response in Arabidopsis [39]; thus, we performed quantitative real-time PCR (qRT-PCR) analysis to verify expression of Medtr1g073320 in rhizobia inoculated roots (Additional file 2: Fig. S6). The result confirms induction of gene expression in infected roots days after rhizobia infection. To establish Medtr1g073320 localisation, C-terminal YFP fusions were introduced in *M. truncatula* roots using *Agrobacterium rhizogenes*-mediated transformation [58]. One-week-old transgenic roots were counterstained with aniline blue to determine callose. Confocal microscope images show Medtr1g073320-YFP in a punctate pattern on the cell periphery, co-localising with callose deposits, which indicates PD targeting (Fig. 7b–i, see also Additional file 2: Fig. S7).

We phenotypically studied these plants to determine the effect of Medtr1g073320-YFP ectopic expression in transgenic roots. Hairy root transgenic Medtr1g073320 plants, grown in soil, look phenotypically similar to transgenics transformed with a control vector, but there is a small significant increase in root and shoot weight (Additional file 2: Figure S8).

**Fig. 8:**
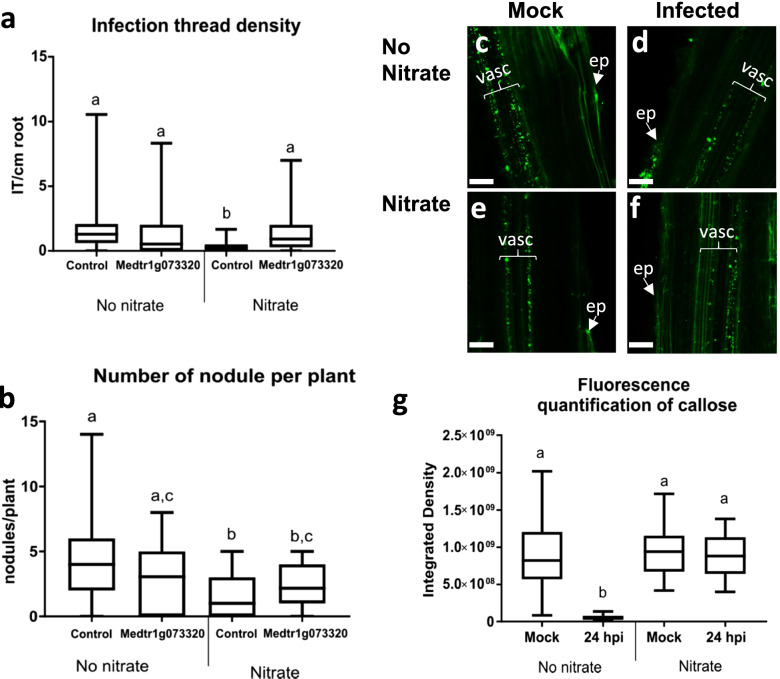
Ectopic Medtr1g073320 expression affects rhizobia infection and callose regulation in nitrate replete conditions. *Medicago truncatula* roots expressing an empty vector (control) or p*35S*-Medtr1g073320 were grown in 0 (no nitrate) or 5 mM nitrate and inoculated with either a mock or rhizobia (infected) liquid culture. Graphs show infection thread density (**a**) and nodule number (**b**) at 7 days post- rhizobia inoculation. Different letters refer to statistically significant differences (*p* <0.05) according to Student’s T-test (see also Additional file 2: Fig. S9). **c-g** Immunofluorescent localization of callose was carried out in p*35S*-Medtr1g073320 roots grown in no nitrate or nitrate replete conditions and inoculated with either a mock or rhizobia culture (infected). Roots were fixed 24 hr post inoculation and callose antibodies were detected with Alexa 488 conjugated antibody (green). Panels **c-f** show representative confocal pictures of each condition. The vascular cylinder (vasc) and the epidermis (ep) are indicated. Scale bars= 50 μm. **g** Integrated density was calculated for each image in a region of interest (ROI) of approximately 100 μm^2^ in at least 3 biological repetitions. Boxes delimit minimum to maximum integrated density values. The central lines refer to the mean. Different letters refer to statistically significant differences (*p* < 0.05, Student’s T-test)

To identify if Medtr1g073320 participates in the regulation of rhizobia infection, as suggested by the gene expression data, hairy roots were transferred to media containing 0- or 5-mM nitrate (KNO_3_) and mock infected or rhizobia (*S. meliloti*) culture as described in the ‘Methods’ section. Nitrate inhibits rhizobia infection and nodulation in *Medicago truncatula* [59]. Seven days after inoculation, infection threads (IT) and nodules were counted in control roots and in Medtr1g073320 overexpressing roots. In no nitrate media, there was no significant difference in infection density or nodule number (Fig. 8a, b). The presence of nitrate reduces both infection and nodulation in control roots, but this effect was not observed in roots ectopically expressing Medtr1g073320 (see also Additional file 2: Figure S9). Comparing control and Medtr1g073320 hairy root transgenics infected with rhizobia in nitrate indicate no changes in root length but increase in infection and nodule number (Figure S9).

To determine the role of callose in this response, immunolocalisation experiments were carried out to compare hairy root transformed with Medtr1g073320 grown in the absence or presence of nitrate (Fig. 8c–g). Callose deposition was measured by quantifying fluorescence values in a set size Region of Interest (ROI). In control roots (transformed with an empty vector), callose is downregulated in response to rhizobia in both nitrate and no nitrate media as reported before [42] (Additional file 2: Figure S9d). Callose was also downregulated 24 hpi with rhizobia in Medtr1g073320 roots grown without nitrate. In contrast, callose accumulation was not significantly different when Medtr1g073320 roots grown in nitrate were inoculated with either mock or a rhizobia culture (Fig. 8e–g).

Altogether, the results indicate a role for Medtr1g073320 in the regulation of callose in response to rhizobia infection in nitrate replete media. These findings also demonstrate the use of the pipeline, implemented in our custom build R script, to predict PD proteins and their function in plant species where experimental PD proteomes are yet unavailable.

## Discussion

Many aspects of PD function and regulation are not well understood despite increasing evidence of the important role these structures play in plant signalling, organ development and response to physiological and environmental cues [1, 40, 60]. Proteomic data is lacking for most plant species due to difficulties in isolating clean PD fractions. Moreover, protein association to PD domains can be quite dynamic, varying in different environmental conditions and tissue types [19, 22, 27]. These factors limit our current knowledge of what proteins, mechanisms and conditions regulate PD properties and function in plant development.

We created a pipeline (PIP1) that exploits the overlap in subfamily composition of existing PD proteomes to generate lists of candidate PD genes (in silico proteomes) for 22 plant species (Additional file 1: Table S2). Expression network analysis of PIP1 identified clusters of co-expressed genes over-represented in PD verified proteins. PIP1 genes in these co-expression clusters are strong candidates for the discovery of new PD regulatory proteins. Their characterisation in the context of PD regulation would significantly improve our knowledge and provide new tools to achieve PD modifications in diverse plant species.

Conditions that regulate the expression of these co-expressed clusters of genes were dissected further in microarray analysis. This led to the identification of osmotic stress and salinity as conditions affecting callose and symplasmic passive transport of GFP in Arabidopsis roots. Combination of PIP1 and co-expression analysis also led to the identification of Medtr1g073320, a PD-located protein co-expressed with MtBG2 and SUNN in *M. truncatula* roots that regulates rhizobia infection, nodulation and callose deposition in nitrate replete conditions. This finding links, for the first time, PD regulation and the mechanism that inhibits the formation of nitrogen-fixing nodules in the presence of nitrate.

Our script works well with all plant species and genes with subfamilies annotated in both PANTHER16 and Ensembl Plant databases. Its applicability will expand as new and updated UniProt Reference Proteomes are added to these databases [44, 45, 61]. For the 40 reference plant genomes included in PANTHER16, family/subfamily annotation coverage is between 60–95% depending on species [45]. A limitation of our pipeline is that genes without a PANTHER classification will not be included in the output.

The PIP1 proteome is classified according to subfamily representation in experimental PD proteomes and according to the presence of SP, GPI or TM domains which are features enriched in PD-verified proteins. For some applications, using family instead of subfamily classifications may be more appropriate. For example, experimental proteome data used as PIP1 input is only available for the dicot species Arabidopsis, tobacco and poplar; thus, using subfamily annotation might not be appropriate to identify orthologues in evolutionary divergent monocots or non-angiosperms species [62]. Using subfamily classifications, we failed to output known PD proteins such as Arabidopsis CRINKLY4 (ACR4) or PDLP5 likely because these subfamilies are low represented or absent in cell cultures (the material used in experimental proteomes). PIP1 prompts users whether to generate candidates based on family or subfamily identifiers. For the studies presented here, we used subfamily classifications, because this significantly reduces the number of candidates identified.

The pipeline was demonstrated to be effective in predicting the poplar proteome showing significant overlap with the experimentally determined PD-enriched fraction. Based on our categorisation, PIP1-A and PIP1-C (proteins with predicted membrane targeting features) are more likely to contain PD localised proteins. Supporting our predictions, lists A and C contained mostly secreted proteins including 36 out of the 60 proteins reported to target PD in *A. thaliana*. Most PD-verified proteins predicted by PIP1 were included in experimentally determined proteomes, but PIP1 also identified proteins characterised in independent studies (Additional file 1: Table S1). For example, the Arabidopsis in silico proteome contains BAM2, the PLASMODESMAL GERMIN-LIKE PROTEIN1 (PDGLP1) and CALNEXIN2 (CNX2), which are not present in any of the experimental proteomes but independently found to localise at PD [2, 5, 15, 24]. Extending the output to family members increased the coverage of known PD proteins to 83.6% but consequently increased the PIP1-A candidate list to over 2000.

Even when using subfamily annotation, the pipeline generates a list of candidates larger than experimental proteomes because expression in cell cultures is not a pre-requisite for gene identification. PIP1-B list contains proteins from subfamilies that appear in multiple proteomes but lack predicted SP, TM or GPI. Overlap between list B and the mobile proteome, recently reported in dodder parasitising *A. thaliana* [51], suggests that these might represent proteins captured while in transit via PD. List B might also include proteins with unusual or poorly predicted membrane targeting features. This is the case for REM1.2, for example, which lacks a predicted SP, GPI or TM and localises to PD independently of the secretory pathway [29, 63, 64]. Further research is required to verify which proportion of list B contains mobile proteins and which contains peripheral membrane proteins.

Besides the obvious reasons of obtaining in silico proteomes as a tool for the identification of new PD proteins, we propose that transcriptomic analysis of the candidate lists predict pathways and conditions affecting PD transport. Here, we showed that osmotic and salinity stresses modify the expression of PD candidates and verified proteins identified in PIP1. Results showing changes in callose and symplasmic transport in the presence of sodium chloride and PEG aligns well with recent publications reporting re-localisation of PD proteins after high salt and mannitol treatment [19, 22]. It is not clear if PD function is affected by changes in the localisation of PD proteins or by changes in turgor pressure or, more likely, by a combination of these [65]. Gene expression interactome networks point to callose as a main regulator of these responses. Callose accumulates at cell walls surrounding PD, restricting symplasmic intercellular transport [36]. We confirmed that callose is induced when seedlings are exposed to 75-mM NaCl and that GFP diffusion rootwards, from the transition zone to the root meristem, is reduced after exposure to 3% PEG or 75-mM NaCl (Fig. 5). Interestingly, active transport of SHR (a transcription factor that determine root ground tissue specification) in a dose-dependent fashion [56] between the stele and the endodermis was not significantly affected in osmotic conditions, but expression and accumulation in the QC were reduced in the presence of 75-mM NaCl. Moreover, roots ectopically expressing PDLP1 (reported to induce callose) are partially insensitive to PEG treatment although able to respond effectively to high NaCl. This finding supports a model in which high salt and drought regulate the expression of PD genes, leading to callose accumulation and altering PD function and root development. Future research will use this knowledge and the PIP1 interactomes to identify molecular components of the pathway underlying this response.

Insight on potential components of the mechanism linking PD and the response to osmotic and salinity stress arises from the analysis of co-expression clusters displaying second order interactions between known callose-modifying enzymes and PIP1 genes (Fig. 3d and Additional file 1: Table S6). This ‘callose interactome’ contains genes encoding cell wall modifiers and receptor/signalling proteins. These include activities such as the multicopper oxidases SKU5 and SKS. Members of this family regulate callose during pollen development [66], but their role at PD is unknown. The interactome also include genes encoding cell wall activities such as pectin de-esterification, hemicellulose modification and AGPs. Our past work identified changes in the structure of pectins surrounding PD clusters (i.e. pit fields) [36] and physical interactions between callose and cellulose likely affecting cell wall properties [67]. How callose regulation influences cell walls architecture and how these modifications contribute to the regulation of PD in response to abiotic stress conditions is still unknown and a topic of interest for further investigation.

PIP1 also predicted the in silico PD proteome in *M. truncatula* and co-expression analysis identified Medtr1g073320, a gene induced in roots infected with the nitrogen-fixing bacteria *S. meliloti*. Our past research indicates that symplasmic communication is enhanced in response to rhizobia infection, and this mechanism regulates nodule formation. So far MtBG2, a callose-degrading enzyme, and the receptor SUNN are the only *M. truncatula* proteins described to associate with PD and both regulate cell-to-cell communication to control nodule number during nitrogen-fixing symbiosis [42, 59]. Using fluorescent fusions, we demonstrated that Medtr1g073320 co-localises with callose at PD and found that its expression affects rhizobia infection, nodulation and callose regulation in nitrate replete conditions. Medtr1g073320 is the first PD located receptor-like protein (PDLP family) identified in *M. truncatula* (and a legume). This finding opens doors for research into the signalling mechanisms mediated by PD, that modulate nitrogen fixing symbiosis and nitrate responses, processes of exceptional importance for sustainable agriculture.

## Conclusions

To summarise, our R-based tool integrates the data obtained from PD proteomes and enable the identification of new PD genes in a variety of plant species. Researchers already use sequence-domain analysis and phylogeny to identify PD components expressed outside cell cultures [18, 28, 42]. Our comparative meta-analysis provides a platform to systematically apply this approach enabling in silico predictions of whole PD proteome based in multiple instead of single experimentation. New information, from proteomics or from independent analysis of PD proteins in diverse plant species, can be added as input to PIP1 to improve in silico predictions. Together with transcriptome analysis, the pipeline becomes a useful tool to identify proteins and conditions affecting PD function. The pipeline can help in prioritising the targets for validation and can predict PD proteins for species where PD proteomic information is not available. The pipeline is publicly accessible and can be easily modified by the user to add new sequenced proteomes and/or experimentally verified genes improving its prediction capabilities and usefulness for the whole plant community.

## Methods

### Plant material and growth conditions

Seeds of *Arabidopsis thaliana* plants WT (Col-0) and transgenic expressing p*SUC2*::GFP [55] or p*35S*::PDLP1:YFP [28] or p*SHR*::SHR:GFP [56] were surface sterilised with ethanol. Control *Arabidopsis Thaliana* Salts (ATS) media was prepared as described by [68] with 0.8% (w/v) agar (Type E, Sigma- Aldrich). When required, plates were prepared using ATS-based media supplemented with 3% (w/v) polyethylene glycol (MW 8000) or with 75 mM NaCl as indicated. Seeds were stratified at 4°C for 4 days before being transferred to long day light conditions (22°C, 16 h day, 150 mE/m^2^/s) for growth. Plant phenotypes and root length were measured at 7 days post-germination. Significant differences were determined using one-way ANOVA (Tukey post hoc test). Media water potential (at 25°C) was estimated by adding the estimated solute potentials of individual medium components based on empirical and modelled data [69–71].

For *p35S::*Medtr1g073320-YFP transgenic roots, Gateway cloning was used to generate the vector following manufacturer’s instruction (Invitrogen, USA). In brief, primers were designed to amplify Medtr1g073320 with linkers compatible for cloning into pDNR221 by BP reaction (Medtr1g0733201-Attb1: GGG GAC AAG TTT GTA CAA AAA AGC AGG CTC CAT GTT TTG ATT CTC TCT CCA; Medtr1g0733201-Attb2: GGG GAC CAC TTT GTA CAA GAA AGC TGG GTA CCA CAA ATC TCT TTC AGC CAA AA). Positive pDNR clones were confirmed by sequencing and used in LR reaction with the destination vector pB7YWG2 [72]. The *p35S::*Medtr1g073320-YFP vector was amplified in *E. coli* and expressed in *Agrobacterium rhizogenes* for transformation. The empty vector (without Medtr1g073320) was transformed alongside and used to generate control transgenic roots.

WT A17 *Medicago truncatula* seeds were lightly scarified with sandpaper, sterilised for 3 min in a 10% sodium hypochlorite solution, washed with water and left, undisturbed in water for 4 h. The seeds were transferred to agar-water plates and left in the dark for 3–7 days at 4°C. Plates were transferred to RT overnight and *A. rhizogenes* cultures (carrying the *p35S-*Medtr1g073320-YFP or the control vector) were used for root transformation as described by [58]. After 2 to 4 weeks, transgenic roots expressing YFP fusions were identified using fluorescent microscopy and selected for confocal imaging and rhizobia infection assays. Composite plants were also transferred to soil and at 44 days after transformation root and shoot weight was measured. Significant differences were determined using a Student’s *T* test.

### PD proteome meta-analysis

PD proteomic data for *A. thaliana*, *Nicotiana benthamiana* and *Populus trichocarpa* were retrieved from their original publications [2–5] and subfamilies annotated based on PANTHER16 [45]. A comprehensive list of experimentally verified PD proteins in *A. thaliana* was assembled by identifying publications reporting protein fusions displaying characteristic punctate localisation in the cell periphery that co-localise with aniline blue or callose deposits when observed using confocal or electron microscopy (Additional file 1: Table S1). The proteomes and list of known PD proteins were incorporated into a pipeline using a custom-built R script deposited in GitHub [43]. Instructions on how to use the pipeline are included in the GitHub repository along with instructions on how to customise pipeline parameters depending on user requirements. Necessary databases, including the PD proteomes, are with the script in the GitHub repository [43].

The script dependencies include the R library ‘biomartr’ [61]. Protein features were predicted using the R library ‘ragp’ [46]. This tool uses SignalP [73, 74] and Phobius [75] to predict SP and PredGPI [76] and NetGPI [77] to predict GPI anchors. The tool ‘ragp’ was also used to predict subcellular localisation via TargetP [74].

Genes were classified as encoding a GPI and/or SP when at least one tool returned true for that feature. TM domain prediction was made using TMHMM and the ENSEMBL database annotation via the R package ‘biomartr’ [47]. Predictions for N-myristoylation, S-farnesylation, S-geranylgeranylation, S-palmitoylation and S-nitrosylation were made using tools available by the Cuckoo workgroup [48–50]. The tools described above were used in protein feature enrichment analysis for the whole *A. thaliana* genome (Araport11). Fisher’s exact test was used to determine statistical significance (cutoff provided in figure legends). To be fully pipeline-compatible for proteome input or to generate candidate gene output, the species must be listed in both Ensembl Plant databases (used by biomartr to retrieve sequence information) and in PANTHER16 (used to retrieve subfamily annotation) [44, 45, 61]. Currently, there are 22 compatible plant species (Additional file 1: Table S2). For non-compatible species, such as *N. benthamiana*, Arabidopsis orthologues can be used. This enables integration in the pipeline of the PD proteome described in [3].

Lists of genes were compared by drawing a Venn or Euler diagram using the R library ‘eulerr’. The significance of the overlap between candidate lists and proteomes was determined using bootstrap analysis. Sets of genes (the same length as a candidate list) and a proteome were randomly sampled from Araport11. The overlap in genes between the samples was recorded and repeated for *n* cycles (*n* = 10,000). Probability (*p*) was calculated as the proportion of cycles that attained an overlap at least as large as observed between the candidate list and the proteome. The size of the overlap by chance was given as the median overlap in random samples over *n* cycles.

### Expression and cluster analysis

The gene correlation dataset ‘Ath-u.c1-0’ was downloaded from the ATTED-II database [52, 78]. Optimal gene order and the corresponding dendrograms were computed using hierarchical clustering. The dendrogram was cut at an optimised height (*h* = 16) that gave a sufficient number/size of clusters (*k* = 151). These processes were performed in base R. Enrichment of genes within clusters from candidate lists and verified genes were determined using pairwise comparisons with Fisher’s exact test (*p*<0.05, holm-adjusted) via the R library ‘rcompanion’.

For network analysis, pairwise correlation data were compiled and exported for *A. thaliana* PD candidates using R. The network of <1000 genes (nodes) and <300,000 interactions (edges) was fed into Cytoscape [53] with correlation set as the edge attribute.

Publicly available microarrays for a subset of conditions were independently analysed. Microarray datasets were downloaded from EBI ArrayExpress [54]. For each experiment, expression data were normalised using the robust multi-array average (RMA) method and log_2_ transformed with the R package ‘oligo’ [79]. Principal component analysis was used to identify and exclude outlier arrays and experiments with insufficient biological replicates. Genes with low levels of expression were filtered out. A design matrix was constructed for each experiment and a linear model applied using the R package ‘limma’ [80]. Differential expression of genes and a multiple comparison correction were determined using empirical bayes statistics via the package ‘limma’ and the results filtered by gene IDs. Heatmaps were constructed using the R package ‘ComplexHeatmap’ [81].

### qRT-PCR expression analysis

To confirm Medtr1g073320 differential expression in response to rhizobia, qRT-PCR experiments were carried out using primers to amplify the target gene and the housekeeping gene ACTIN. *Medicago truncatula* roots were spot inoculated and a window of 1 cm of root containing the inoculation point was collected at different time points and immediately frozen in liquid nitrogen. RNA was extracted using the RNeasy Plant Mini Kit following the manufacturer’s instructions (Qiagen). Quality and concentration of RNA were evaluated by electrophoresis and NanoDrop® Spectrometer ND-1000.

One microgram of RNA was used per sample to synthesise cDNA using SuperScript II (ThermoFisher) and Oligo dT following manufacturer’s protocol. cDNA was used in standard PCR reactions to semi-quantify transcription with the following primers: RTPCR-MtACTIN-Fw: GAC AAT GGA ACT GGA ATG GTG; RTPCR-MtACTIN-Rv: CAA TAC CGT GCT CAA TGG GG; RTPCR-Medtr1g0733201-Fw: GGT TCC AAA GGG TGG TCA CT; RTPCR-Medtr1g0733201-Rv: GGC CTC CAC AGT AAA CCA TAT.

Real-time PCR was carried out in a CFX ConnectTM Real-Time PCR Detection System using CFX96 TouchTM programme for recording the results (Bio-Rad). SYBR green was used for quantification of dsDNA synthesis during amplification. The relative gene expression levels were calculated using the comparative Ct (𝚫𝚫Ct) method [82], where Ct represents the threshold cycle. The qRT-PCR in Fig. S6 represents mean values of three replicas +/− SD calculated as described [82].

### Phylogenetic tree

Protein sequences containing the ‘Domain of Unknown Function 26’ (DUF26) domain and a transmembrane domain were isolated from *M. truncatula* and *A. thaliana* as described in [41]. To eliminate redundancies, all sequences isolated were aligned using Muscle 58 and phylogenetic trees calculated using Bayesian inference of phylogeny algorithm. The best model under the Akaike information criterion was LG+G. Majority-rule consensus trees convergence was reached after 90,000 generations. The trees were visualised using the software Figtree [83] and edited using TreeGraph2 [84].

### Confocal microscopy: symplasmic transport, callose detection and protein localization

To determine changes in symplasmic transport, seedlings of *A. thaliana* expressing either *pSUC2:*:GFP or p*SHR*::SHR-GFP were mounted on glass slides in 10 μg/ml propidium iodide (Sigma-Aldrich). Root tips were imaged using an LSM 800 upright confocal microscope (Zeiss, Germany). A 488 nm (excitation laser) was used to capture GFP. Profiles of fluorescence were determined using line and profiling tools in ImageJ. Lateral profiles were taken across the transition zone and rootward profiles started from the basal/apical meristem transition zone ending 150 μm towards the root tip. The fluorescence of lateral and rootward profiles were scaled between 0 and 1. Fluorescence across each lateral profile was binned (bins = 100) to compensate for small differences in root width. Fluorescence profiles of at least 6 plants per treatment were aggregated by calculating the mean (±SD) for position along the profile and plotted.

For callose staining, decolourised aniline blue solution was used following published protocols [85]. Alternatively, aniline blue fluorochrome (Biosupplies, Australia diluted as described in the catalogue in 0.1 M K_3_PO_4_ (pH 12)) was used. Aniline blue staining was captured with 405 nm excitation and emission at 463 nm.

Immunolocalisation was performed using callose monoclonal antibodies (Biosupplies, Australia, diluted as described in the catalogue) in tissues sections as described before [86]. The Biosupplies callose antibody was detected with secondary antimouse-Alexa 488 (Invitrogen, diluted as described by manufacturers) using 488 nm excitation laser in a confocal microscope (LSM-880, Zeiss). Note that quantification was performed in a pool of z-stack images, quantifying several sections of the same root.

To determine Medtr1g073320 protein localization, *M. truncatula* roots expressing p*35S*::Medtr1g073320-YFP were counterstained with aniline blue fluorochome and visualised using a confocal microscope (LSM-880, Zeiss) with excitation laser 561 nm (YFP) and 405 nm (aniline blue). Transmission light images were taken to reveal co-localization in cell walls.

### Rhizobia inoculation and phenotypic analysis

The rhizobia strain *Sinorhizobium meliloti* Sm 1021-lacZ (pXLGD4 lacZ reporter) was used to inoculate transgenic roots expressing p*35S*::Medtr1g073320-YFP. Transgenic *M. truncatula* roots were transferred to square plates containing buffered nodulation medium (with 1μM AVG) and no nitrate or nitrate (5mM KNO_3_). Roots were flooded with a suspension of *Sm 1021-lacZ* OD_600_ 0.05) in 10 mM MgCl_2_ as described before [42]. Nodules, infection threats and infection pockets, were stained with the X-Gal substrate (5-bromo-4-chloro-3-indolyl-b-D galactopyranoside, Thermo Fisher Scientific) and counted per centimetre of root (density) using a Zeiss Axio Scope.A1 and imaged using an Olympus -BH2 fitted with a camera. The data was analysed using Microsoft Excel and R Statistical packages. All phenotyping data were analysed for normality using D’Agostino Pearson omnibus normality test. Phenotyping data regarded to be suitably normally distributed was analysed by one-way ANOVA (with Tukey post hoc test) or Students *t* test unless unequal variance between treatments could not be assumed. In which case, pairwise Welch’s *t* test (two-tailed, Holm corrected) was also used to determine significant differences. Differences were referred to as significant when *p*-values <0.05.

## Supplementary Information

**Additional file 1: Table S1**. *Arabidopsis thaliana* proteins reported to localize at PD. **Table S2**. Plant species compatible with PIP1. List of plant species listed in both PANTHER16 and Ensembl Plant databases at the time of publication. **Table S3**. PANTHER16 subfamilies represented in experimental proteomes. **Table S4**. in silico PD proteome for poplar generated with PIP. **Table S5**. in silico PD proteome for A. thaliana generated with PIP1. **Table S6**. Genes identified in clusters 87 and 100 of the callose interactome shown in Fig. 3d. **Table S7**. Description of the microarrays used in this study. **Table S8**. in silico PD proteome for *Medicago truncatula* using PIP1.

**Additional file 2: Figure S1.** Predictions of membrane targeting features in verified PD proteins. **Figure S2.** Overlap between the experimental PD proteome for poplar and the predicted PIP1 proteome using an abridged pipeline. **Figure S3**. Expression analysis of Arabidopsis thaliana PD candidates and PD verified genes in abiotic and biotic stress transcriptomes. **Figure S4.** Aniline blue staining reveals callose deposits in Arabidopsis root exposed to PEG and NaCl. **Figure S5**. Ectopic expression of the callose regulatory protein PDLP1 restricts root growth and response to 3% PEG. **Figure S6.** Medtr1g073320 is a PDLP- family member upregulated upon rhizobia inoculation in *Medicago truncatula* roots. **Figure S7.** Medtr1g073320 localizes with callose at plasmodesmata. **Figure S8.** Medtr1g073320 overexpression improves root and shoot weight. **Figure S9.** Medtr1g073320 regulates rhizobia infection and nodulation in full-nitrate conditions.

## Data Availability

PIP1, the R-based pipeline described here is available for download along with associated databases from GitHub repository [43] upon publication. The Medtr1g073320-YFP construct will be provided upon request. p*SUC2*-GFP [55], p*SHR*::SHR-GFP [56] and PDLP1OE [28] seeds were obtained from the corresponding author of the original publication reporting these. The co-expression dataset (‘Ath-u.c1-0’) which support the conclusions of this article are available in ATTEDII from https://atted.jp/download/Ath-u.c1-0/coex [52, 78]. Transcriptomic datasets supporting the conclusions of this article are available from EBI ArrayExpress (accessions listed in Additional file 1: Table. S7 and relevant data citations given in the main reference list [87–145]) [54]. All other datasets supporting the conclusions of this article are contained either in Additional file 1 or are deposited in the University of Leeds Data Repository (10.5518/1155) [146].

## Abbreviations

ACR4: *A thaliana* CRINKLY 4
AGP: ARABINOGALACTAN PROTEIN
AtBG_PPAP: *A thaliana* ß-1,3-GLUCANASE_PUTATIVE PD-ASSOCIATED PROTEIN
ATS: *Arabidopsis Thaliana* Salts
AU: Arbitrary units
AVG: Aminoethoxy vinyl glycine
BAM2: BARELY ANY MERISTEM 2
BG: ß-(1,3)–GLUCANASE
CALS: CALLOSE SYNTHASE
CNX2: CALNEXIN 2
DPT: Days-post transfer
DT: Desmotubule
DUF26: Domain of Unknown Function 26
ER: Endoplasmic reticulum
FRET-FLIM: Förster Resonance Energy Transfer by Fluorescence Lifetime Imaging Microscopy
GAUT: GALACTURONOSYLTRANSFERASES
GFP: GREEN FLUORESCENT PROTEIN
GPI: Glycosylphosphatidylinositol
IT: Infection thread
LYM2: LYSIN MOTIF DOMAIN-CONTAINING GPI-ANCHORED PROTEIN 2
MCTP: MULTIPLE C2 DOMAIN AND TRANSMEMBRANE REGION PROTEIN
MtBG2: *Medicago truncatula* ß-(1,3)–GLUCANASE 2
OD_600_: Optical density at 600 nm
PD: Plasmodesmata
PDBG: PD-LOCATED β-(1,3)–GLUCANASE
PDCB: PLASMODESMATA CALLOSE BINDING PROTEIN
PDGLP1: PLASMODESMAL GERMIN-LIKE PROTEIN 1
PDLP: PLASMODESMATA-LOCATED PROTEIN
PG: POLYGALACTURONASE
PEG: Polyethylene glycol
PI: Propidium iodide
PIP1: Plasmodesmata in silico proteome 1
PM: Plasma membrane
PZ: Proliferation zone
QC: Quiescent centre
REM1.2: REMORIN 1.2
RMA: Robust multi-array
ROI: Region of interest
RT: Room temperature
qRT-PCR: Quantitative real-time polymerase chain reaction
SD: Standard deviation
SDM: Standard deviation of the mean
SKS: SKU5-SIMILAR
SHR: SHORTROOT
SP: Signal peptide
SRF: STRUBBELIG-receptor family
SUC2: SUCROSE SYMPORTER 2
SUNN: SUPER NUMERIC NODULES
TM: Transmembrane domain
TZ: Transition zone
UV: Ultraviolet
WT: Wild-type
X-Gal: 5-Bromo-4-chloro-3-indolyl-b-D galactopyranoside
XTH: XYLOGLUCAN ENDOTRANSGLUCOSYLASE/HYDROLASES
YFP: YELLOW FLUORESCENT PROTEIN
