# Supplementary material for: A comparative meta-proteomic pipeline for the identification of plasmodesmata proteins and regulatory conditions in diverse plant species
Source: BMC Biol. 2022 Jun 2;20:128. doi: 10.1186/s12915-022-01331-1 (PMC9164936; doi:10.1186/s12915-022-01331-1)
Supplement: Supplementary file 2 — Additional file 2: Figure S1. Predictions of membrane targeting features in verified PD proteins. Figure S2. Overlap between the experimental PD proteome for poplar and the predicted PIP1 proteome using an abridged pipeline. Figure S3. Expression analysis of Arabidopsis thaliana PD candidates and PD verified genes in abiotic and biotic stress transcriptomes. Figure S4. Aniline blue staining reveals callose deposits in Arabidopsis root exposed to PEG and NaCl. Figure S5. Ectopic expression of the callose regulatory protein PDLP1 restricts root growth and response to 3% PEG. Figure S6. Medtr1g073320 is a PDLP- family member upregulated upon rhizobia inoculation in Medicago truncatula roots. Figure S7. Medtr1g073320 localizes with callose at plasmodesmata. Figure S8. Medtr1g073320 overexpression improves root and shoot weight. Figure S9. Medtr1g073320 regulates rhizobia infection and nodulation in full-nitrate conditions. [file 12915_2022_1331_MOESM2_ESM.pptx]

## Slide 1
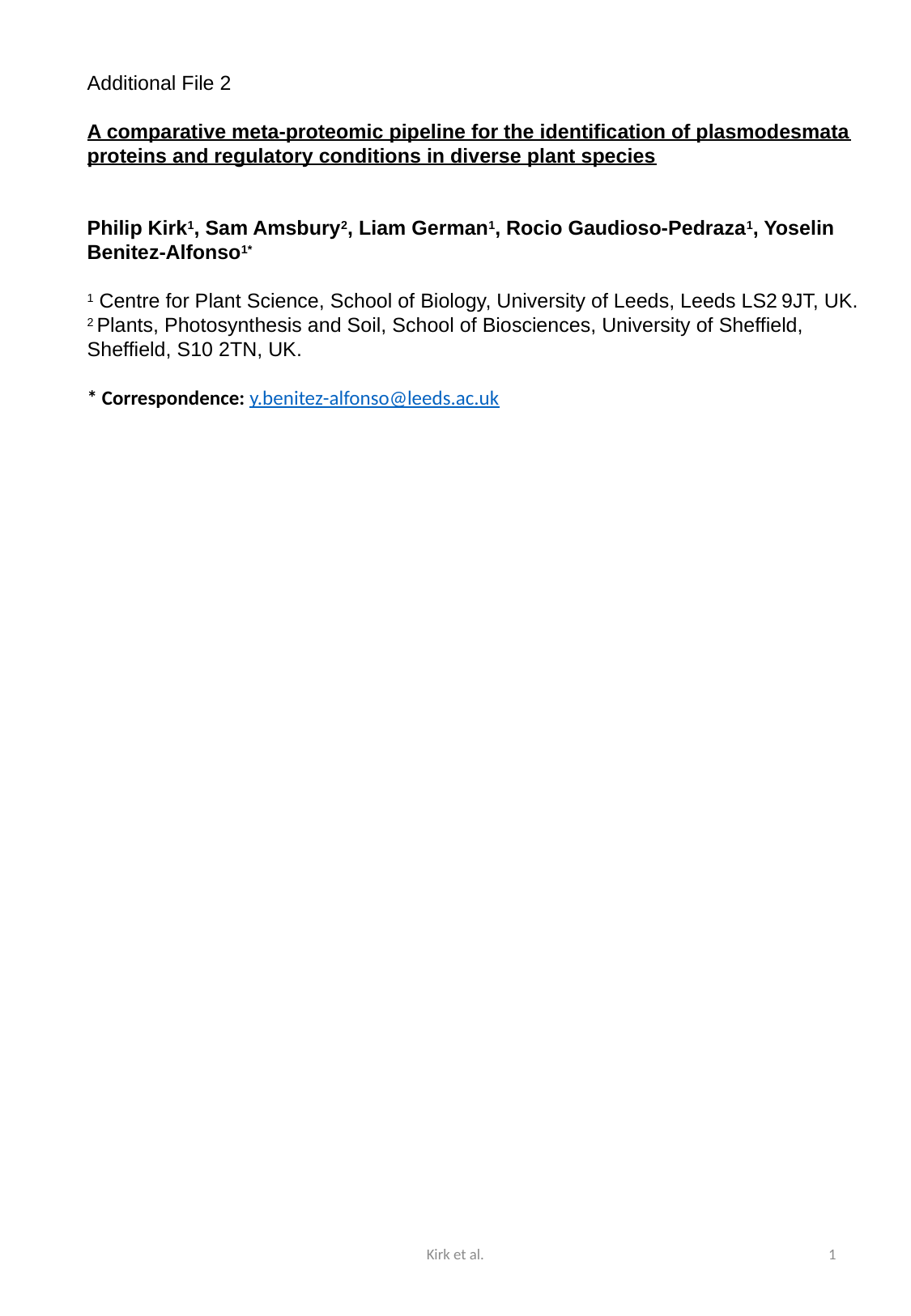

Additional File 2
A comparative meta-proteomic pipeline for the identification of plasmodesmata proteins and regulatory conditions in diverse plant species
Philip Kirk1, Sam Amsbury2, Liam German1, Rocio Gaudioso-Pedraza1, Yoselin Benitez-Alfonso1*
1 Centre for Plant Science, School of Biology, University of Leeds, Leeds LS2 9JT, UK.
2 Plants, Photosynthesis and Soil, School of Biosciences, University of Sheffield, Sheffield, S10 2TN, UK.
* Correspondence: y.benitez-alfonso@leeds.ac.uk
Kirk et al.
1

## Slide 2
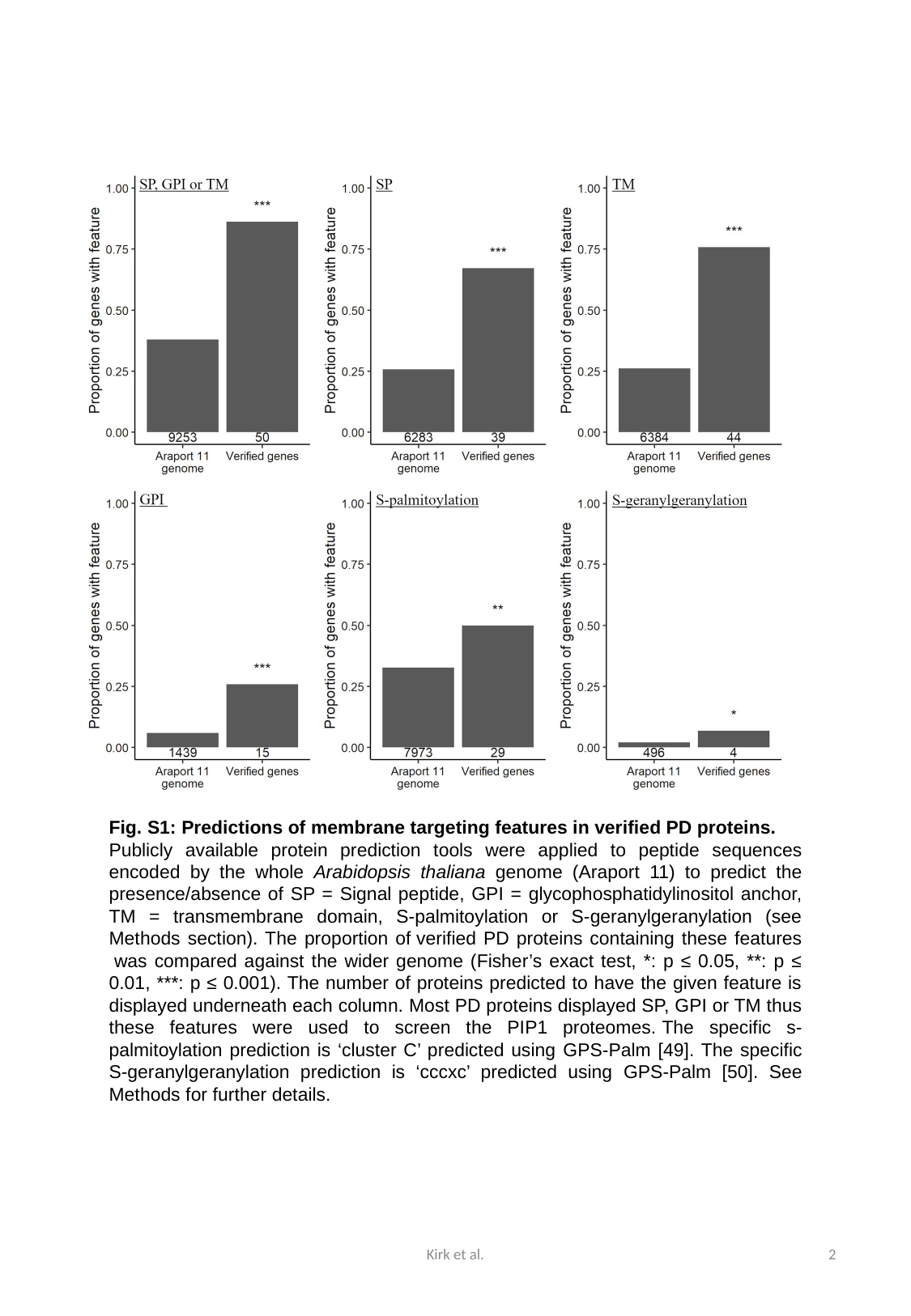

Fig. S1: Predictions of membrane targeting features in verified PD proteins.
Publicly available protein prediction tools were applied to peptide sequences encoded by the whole Arabidopsis thaliana genome (Araport 11) to predict the presence/absence of SP = Signal peptide, GPI = glycophosphatidylinositol anchor, TM = transmembrane domain, S-palmitoylation or S-geranylgeranylation (see Methods section). The proportion of verified PD proteins containing these features  was compared against the wider genome (Fisher’s exact test, *: p ≤ 0.05, **: p ≤ 0.01, ***: p ≤ 0.001). The number of proteins predicted to have the given feature is displayed underneath each column. Most PD proteins displayed SP, GPI or TM thus these features were used to screen the PIP1 proteomes. The specific s-palmitoylation prediction is ‘cluster C’ predicted using GPS-Palm [49]. The specific S-geranylgeranylation prediction is ‘cccxc’ predicted using GPS-Palm [50]. See Methods for further details.
Kirk et al.
2

## Slide 3
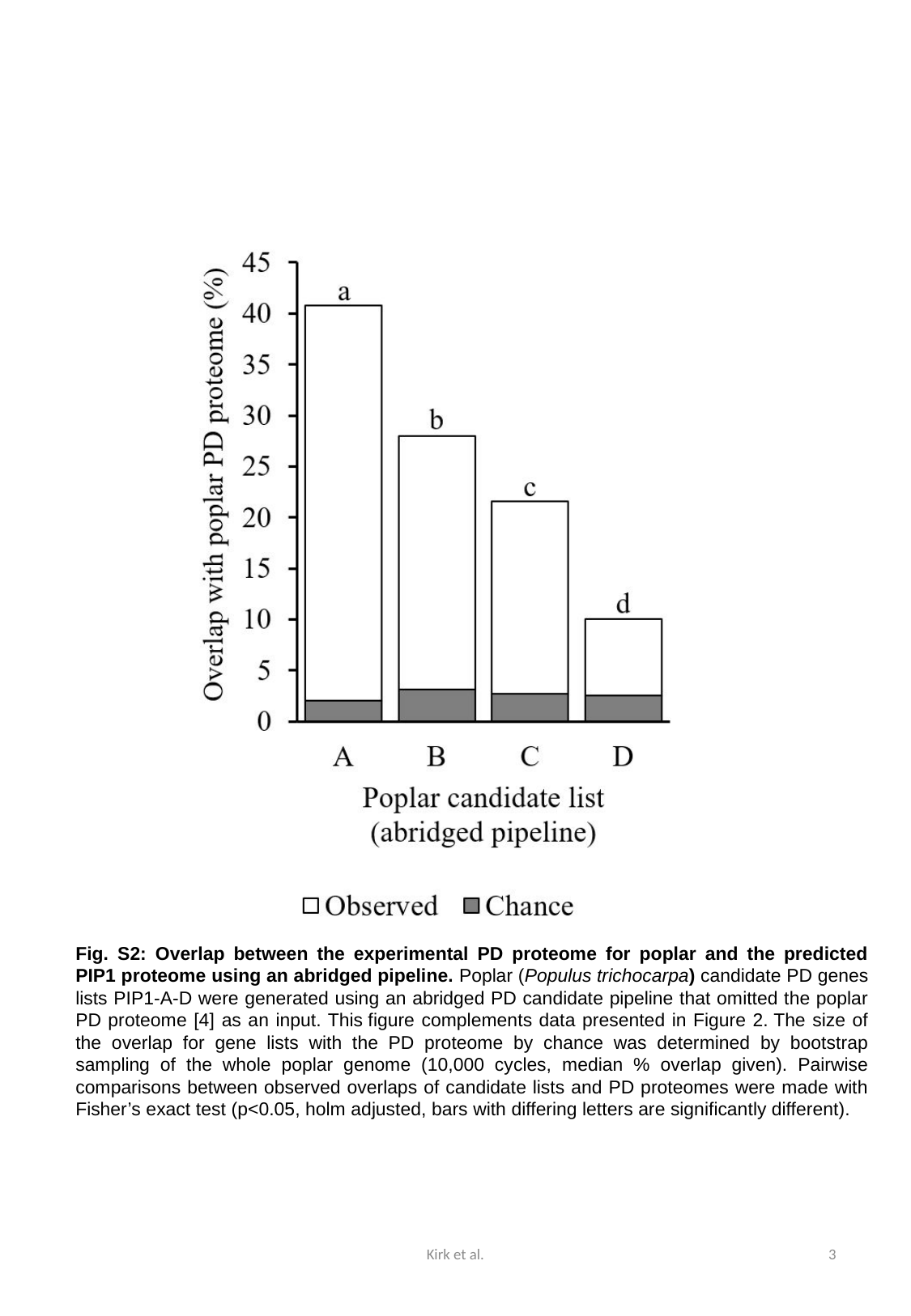

Fig. S2: Overlap between the experimental PD proteome for poplar and the predicted PIP1 proteome using an abridged pipeline. Poplar (Populus trichocarpa) candidate PD genes lists PIP1-A-D were generated using an abridged PD candidate pipeline that omitted the poplar PD proteome [4] as an input. This figure complements data presented in Figure 2. The size of the overlap for gene lists with the PD proteome by chance was determined by bootstrap sampling of the whole poplar genome (10,000 cycles, median % overlap given). Pairwise comparisons between observed overlaps of candidate lists and PD proteomes were made with Fisher’s exact test (p<0.05, holm adjusted, bars with differing letters are significantly different).
Kirk et al.
3

## Slide 4
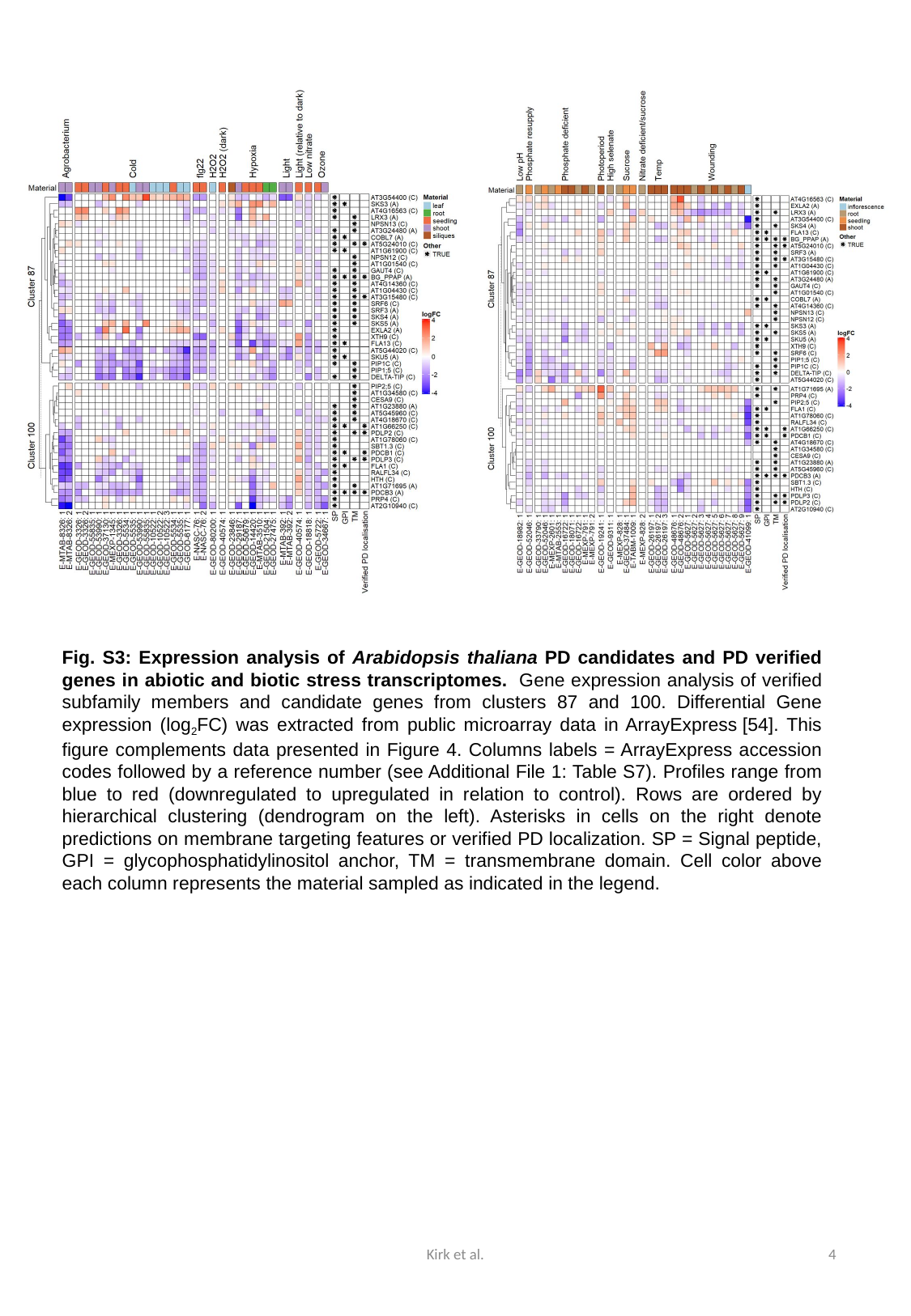

Fig. S3: Expression analysis of Arabidopsis thaliana PD candidates and PD verified genes in abiotic and biotic stress transcriptomes.  Gene expression analysis of verified subfamily members and candidate genes from clusters 87 and 100. Differential Gene expression (log2FC) was extracted from public microarray data in ArrayExpress [54]. This figure complements data presented in Figure 4. Columns labels = ArrayExpress accession codes followed by a reference number (see Additional File 1: Table S7). Profiles range from blue to red (downregulated to upregulated in relation to control). Rows are ordered by hierarchical clustering (dendrogram on the left). Asterisks in cells on the right denote predictions on membrane targeting features or verified PD localization. SP = Signal peptide, GPI = glycophosphatidylinositol anchor, TM = transmembrane domain. Cell color above each column represents the material sampled as indicated in the legend.
Kirk et al.
4

## Slide 5
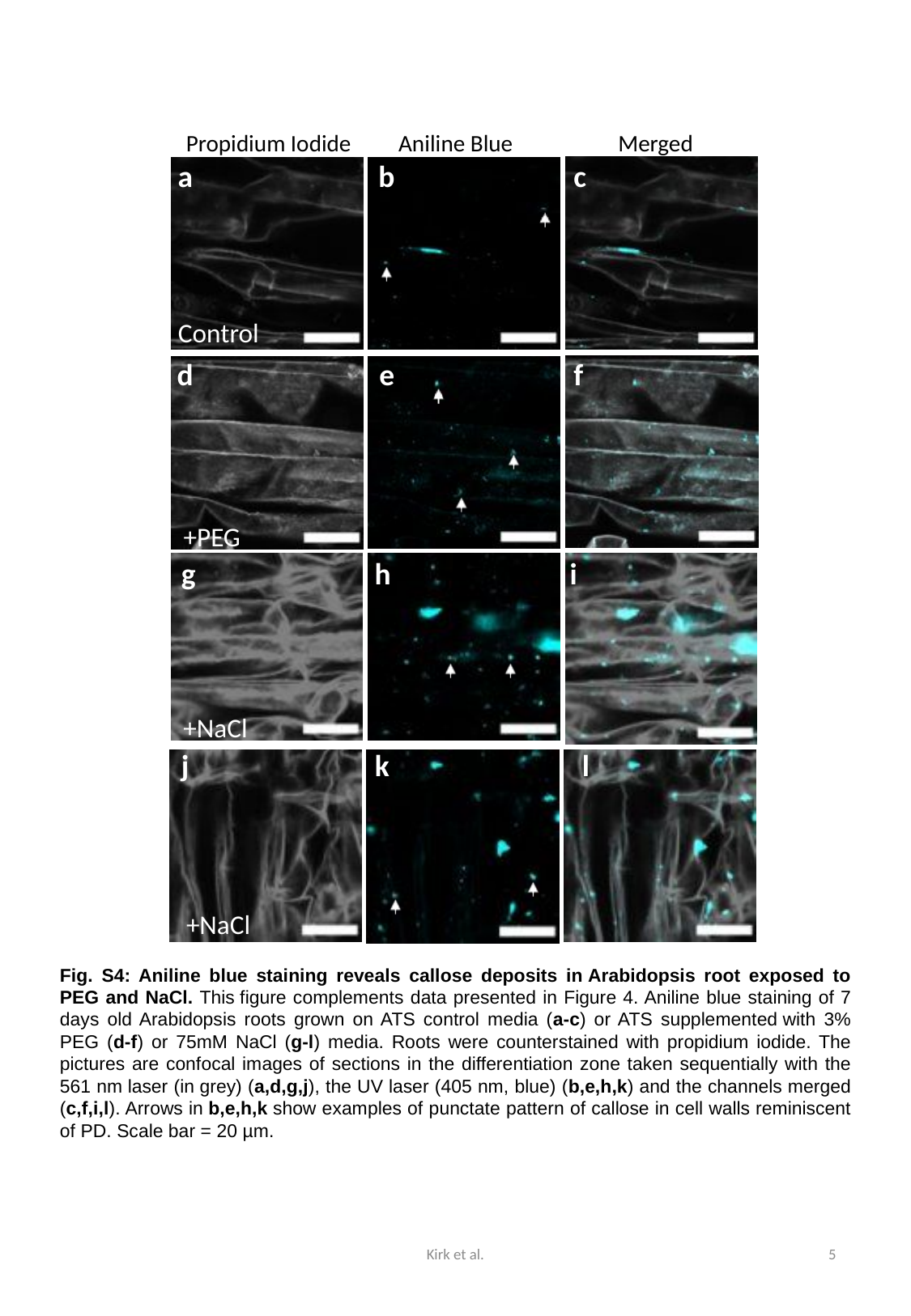

Propidium Iodide         Aniline Blue                    Merged
a                           b                          c
Control
d                           e                          f
+PEG
g                          h                          i
+NaCl
j                           k                            l
+NaCl
Fig. S4: Aniline blue staining reveals callose deposits in Arabidopsis root exposed to PEG and NaCl. This figure complements data presented in Figure 4. Aniline blue staining of 7 days old Arabidopsis roots grown on ATS control media (a-c) or ATS supplemented with 3% PEG (d-f) or 75mM NaCl (g-l) media. Roots were counterstained with propidium iodide. The pictures are confocal images of sections in the differentiation zone taken sequentially with the 561 nm laser (in grey) (a,d,g,j), the UV laser (405 nm, blue) (b,e,h,k) and the channels merged (c,f,i,l). Arrows in b,e,h,k show examples of punctate pattern of callose in cell walls reminiscent of PD. Scale bar = 20 µm.
Kirk et al.
5

## Slide 6
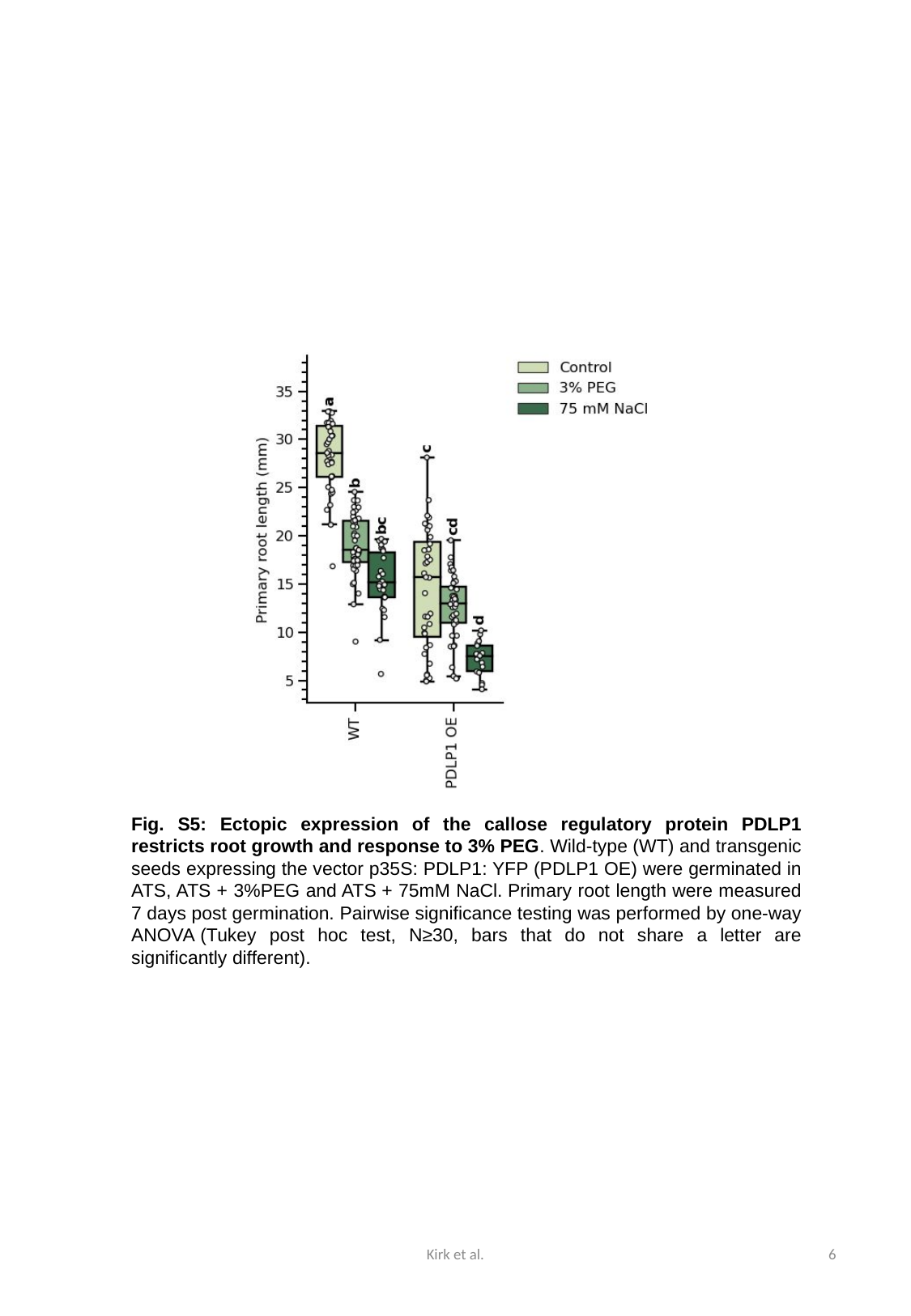

Fig. S5: Ectopic expression of the callose regulatory protein PDLP1 restricts root growth and response to 3% PEG. Wild-type (WT) and transgenic seeds expressing the vector p35S: PDLP1: YFP (PDLP1 OE) were germinated in ATS, ATS + 3%PEG and ATS + 75mM NaCl. Primary root length were measured 7 days post germination. Pairwise significance testing was performed by one-way ANOVA (Tukey post hoc test, N≥30, bars that do not share a letter are significantly different).​
Kirk et al.
6

## Slide 7
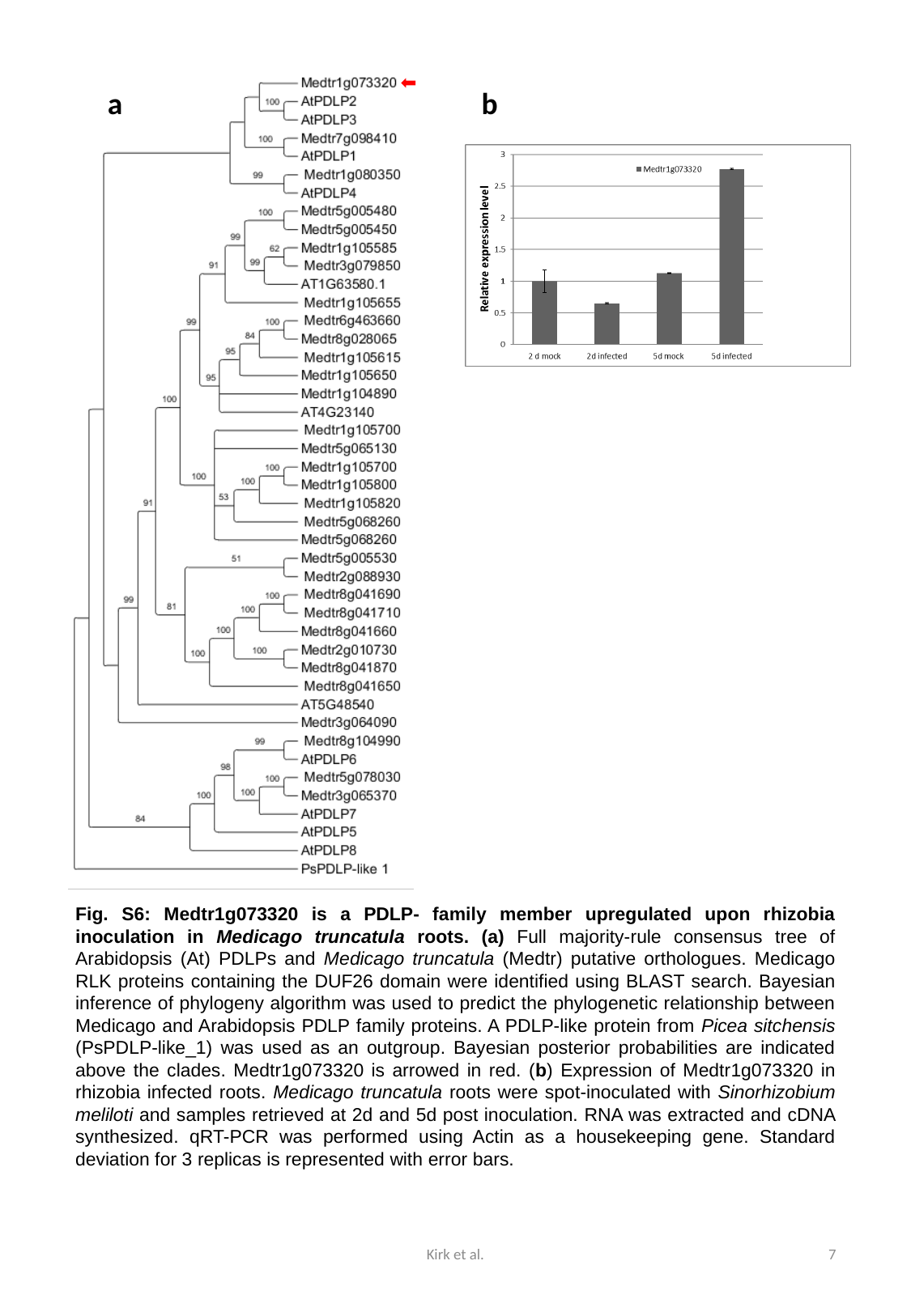

a
b
Fig. S6: Medtr1g073320 is a PDLP- family member upregulated upon rhizobia inoculation in Medicago truncatula roots. (a) Full majority-rule consensus tree of Arabidopsis (At) PDLPs and Medicago truncatula (Medtr) putative orthologues. Medicago RLK proteins containing the DUF26 domain were identified using BLAST search. Bayesian inference of phylogeny algorithm was used to predict the phylogenetic relationship between Medicago and Arabidopsis PDLP family proteins. A PDLP-like protein from Picea sitchensis (PsPDLP-like_1) was used as an outgroup. Bayesian posterior probabilities are indicated above the clades. Medtr1g073320 is arrowed in red. (b) Expression of Medtr1g073320 in rhizobia infected roots. Medicago truncatula roots were spot-inoculated with Sinorhizobium meliloti and samples retrieved at 2d and 5d post inoculation. RNA was extracted and cDNA synthesized. qRT-PCR was performed using Actin as a housekeeping gene. Standard deviation for 3 replicas is represented with error bars.
Kirk et al.
7

## Slide 8
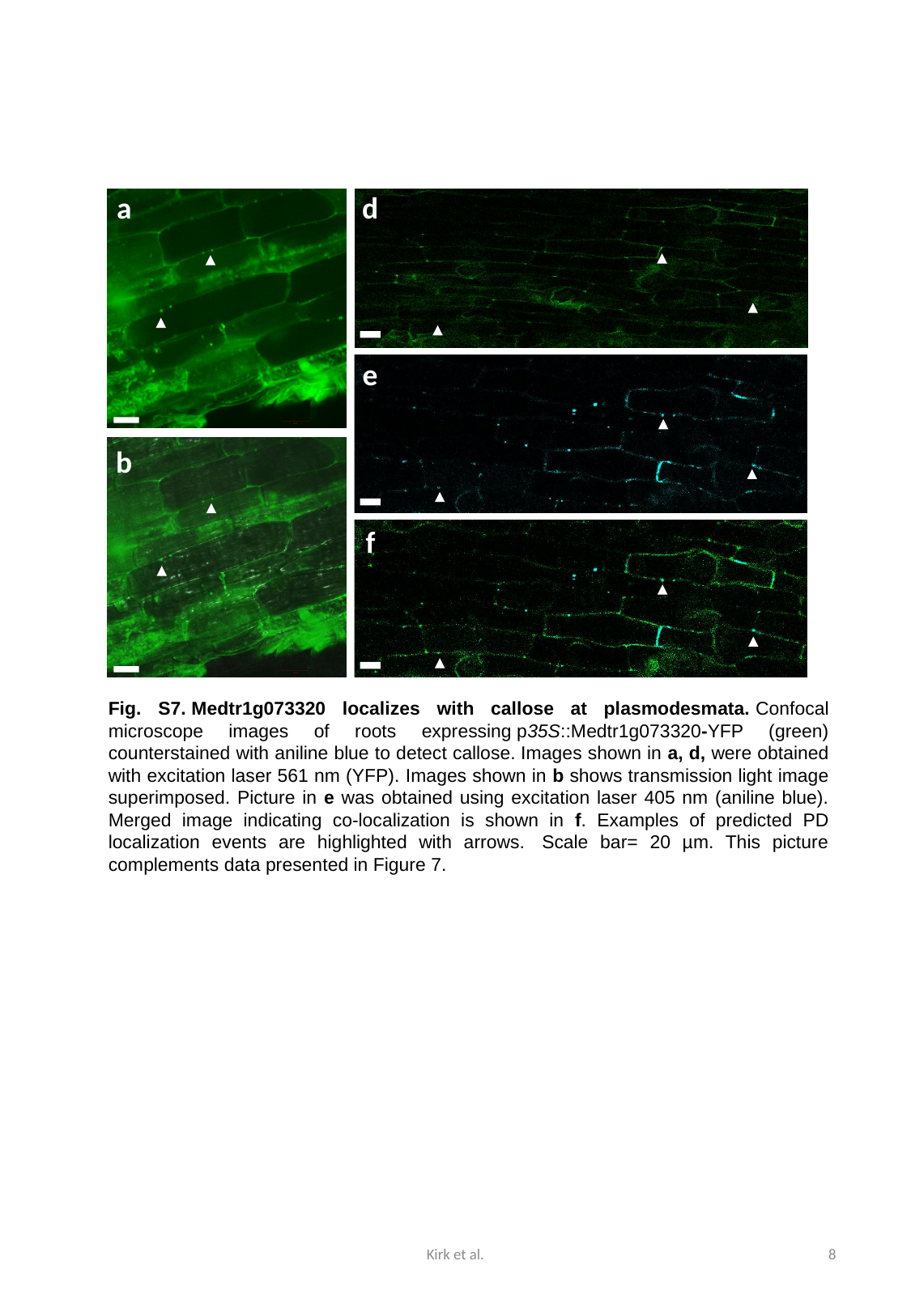

a
d
e
b
f
Fig. S7. Medtr1g073320 localizes with callose at plasmodesmata. Confocal microscope images of roots expressing p35S::Medtr1g073320-YFP (green) counterstained with aniline blue to detect callose. Images shown in a, d, were obtained with excitation laser 561 nm (YFP). Images shown in b shows transmission light image superimposed. Picture in e was obtained using excitation laser 405 nm (aniline blue). Merged image indicating co-localization is shown in f. Examples of predicted PD localization events are highlighted with arrows.  Scale bar= 20 µm. This picture complements data presented in Figure 7.
Kirk et al.
8

## Slide 9
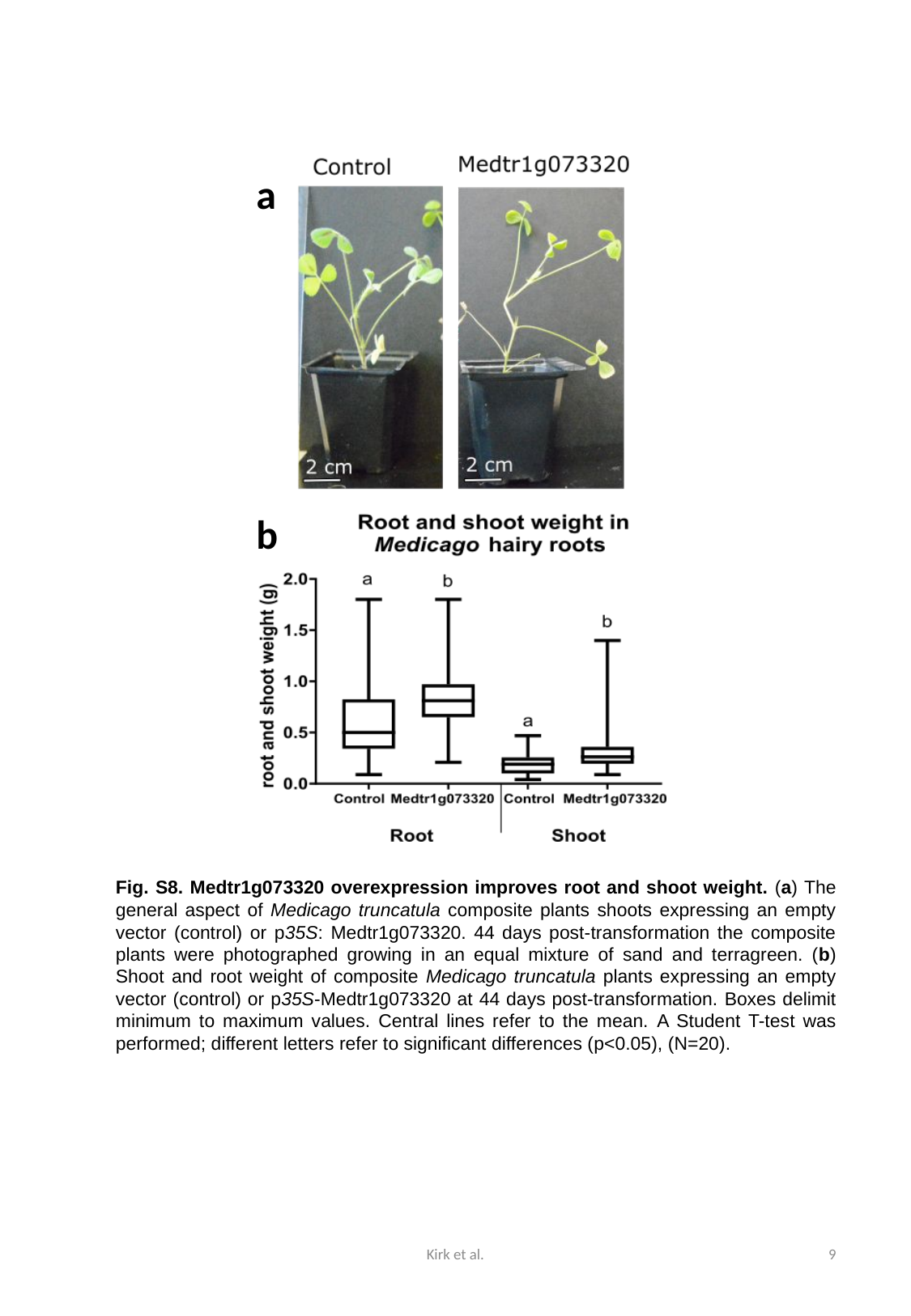

a
b
Fig. S8. Medtr1g073320 overexpression improves root and shoot weight. (a) The general aspect of Medicago truncatula composite plants shoots expressing an empty vector (control) or p35S: Medtr1g073320. 44 days post-transformation the composite plants were photographed growing in an equal mixture of sand and terragreen. (b) Shoot and root weight of composite Medicago truncatula plants expressing an empty vector (control) or p35S-Medtr1g073320 at 44 days post-transformation. Boxes delimit minimum to maximum values. Central lines refer to the mean. A Student T-test was performed; different letters refer to significant differences (p<0.05), (N=20).
Kirk et al.
9

## Slide 10
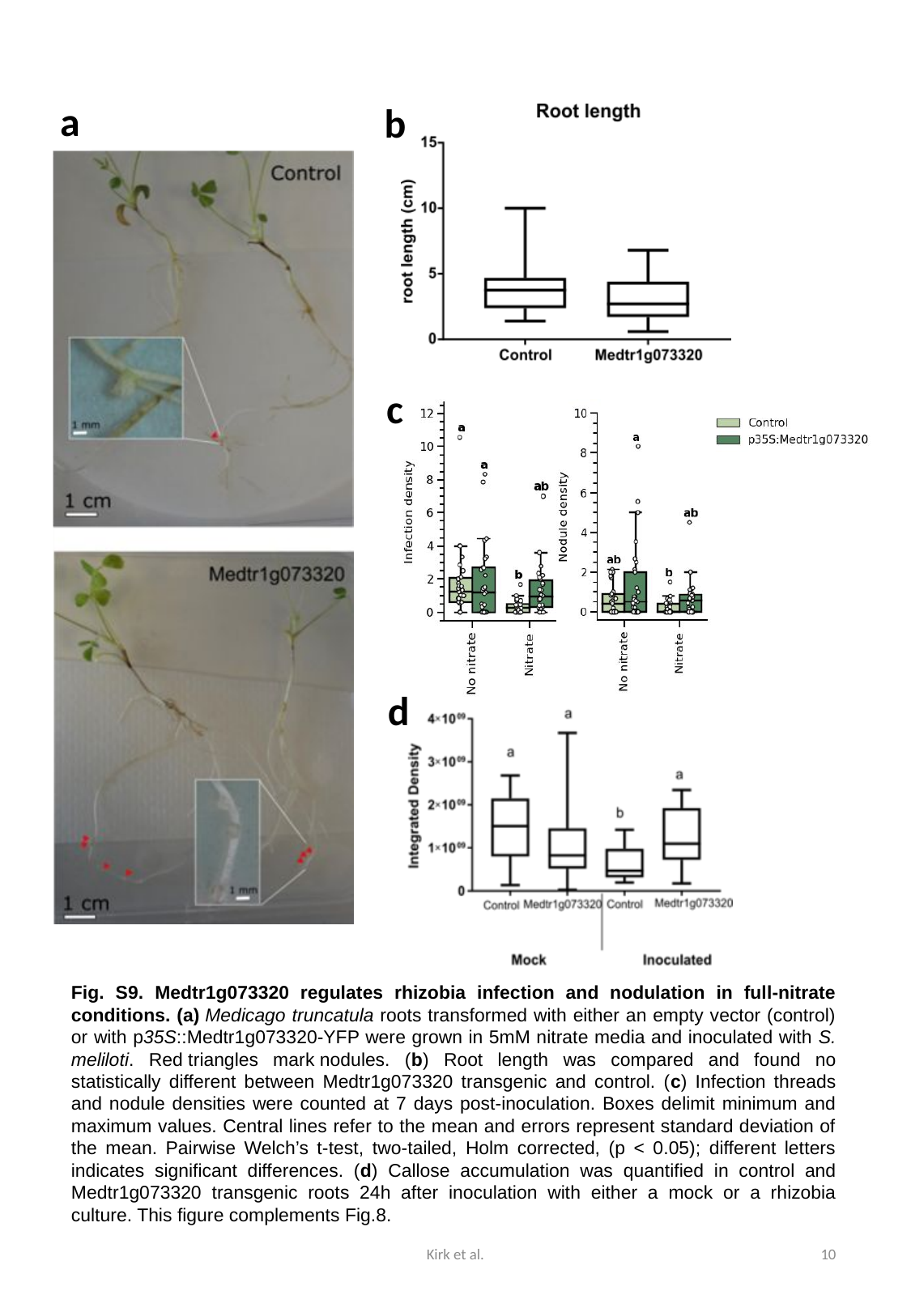

a
b
c
d
Fig. S9. Medtr1g073320 regulates rhizobia infection and nodulation in full-nitrate conditions. (a) Medicago truncatula roots transformed with either an empty vector (control) or with p35S::Medtr1g073320-YFP were grown in 5mM nitrate media and inoculated with S. meliloti. Red triangles mark nodules. (b) Root length was compared and found no statistically different between Medtr1g073320 transgenic and control. (c) Infection threads and nodule densities were counted at 7 days post-inoculation. Boxes delimit minimum and maximum values. Central lines refer to the mean and errors represent standard deviation of the mean. Pairwise Welch’s t-test, two-tailed, Holm corrected, (p < 0.05); different letters indicates significant differences. (d) Callose accumulation was quantified in control and Medtr1g073320 transgenic roots 24h after inoculation with either a mock or a rhizobia culture. This figure complements Fig.8.
Kirk et al.
10
